# Three Molecular Markers Show No Evidence of Population Genetic Structure in the Gouldian Finch (*Erythrura gouldiae*)

**DOI:** 10.1371/journal.pone.0167723

**Published:** 2016-12-09

**Authors:** Peri E. Bolton, Andrea J. West, Adam P. A. Cardilini, Jennalee A. Clark, Kimberley L. Maute, Sarah Legge, James Brazill-Boast, Simon C. Griffith, Lee A. Rollins

**Affiliations:** 1 Department of Biological Sciences, Macquarie University, Sydney, New South Wales, Australia; 2 Centre for Integrative Ecology, School of Life and Environmental Sciences, Deakin University, Geelong, Victoria, Australia; 3 Institute of Conservation Biology and Environmental Management, University of Wollongong, Wollongong, New South Wales, Australia; 4 Australian Wildlife Conservancy, Perth, Western Australia, Australia; National Cheng Kung University, TAIWAN

## Abstract

Assessment of genetic diversity and connectivity between regions can inform conservation managers about risk of inbreeding, potential for adaptation and where population boundaries lie. The Gouldian finch (*Erythrura gouldiae*) is a threatened species in northern Australia, occupying the savannah woodlands of the biogeographically complex monsoon tropics. We present the most comprehensive population genetic analysis of diversity and structure the Gouldian finch using 16 microsatellite markers, mitochondrial control region and 3,389 SNPs from genotyping-by-sequencing. Mitochondrial diversity is compared across three related, co-distributed finches with different conservation threat-statuses. There was no evidence of genetic differentiation across the western part of the range in any of the molecular markers, and haplotype diversity but not richness was lower than a common co-distributed species. Individuals within the panmictic population in the west may be highly dispersive within this wide area, and we urge caution when interpreting anecdotal observations of changes to the distribution and/or flock sizes of Gouldian finch populations as evidence of overall changes to the population size of this species.

## Introduction

Robust estimates of population parameters, such as size and connectivity, are of vital importance to effective conservation and wildlife management. Connectivity describes the movement of individuals, genes and behaviour between regions or groups of individuals, and the degree of connectivity can be used to define populations in a genetic and demographic sense [1]. There is a long history of directly assessing total population size, population growth rates and regional connectivity using methods such as visual observation, mark-recapture and radio tracking [2,3]. These methods are not always logistically feasible or reliable in species that have cryptic behaviour, inhabit remote areas, have large geographic distributions, or persist at low densities [3]. Population size and connectivity estimates derived from these methods may be additionally confounded when individuals or groups are highly mobile (e.g. nomadic or migratory), or numbers fluctuate seasonally [4]. In these situations, population genetics may fill knowledge gaps or improve estimates of population size and connectivity.

The Gouldian finch (*Erythrura gouldiae*) is one such species that could benefit from population genetic investigation, because the absence of robust population estimates has hindered conservation decision making. In the 1970s, Gouldian finch populations experienced strong declines (up to 87%), and became restricted to a few small areas in Western Australia and Northern Territory, and were virtually extirpated from Queensland [5,6]. First identified as Threatened by IUCN Red List in 1988, the Gouldian finch was recently down-listed to Near Threatened by the IUCN, based on population data compiled from largely *ad hoc* observations [7]. Many of these observations come from resident or bird watcher observations that report large flocks (400 and >1000) in geographically distant locations [7]. These observations are unreliable for estimation of total population size because observations are not carried out systematically throughout the range, and occur mostly in the accessible dry season when birds aggregate around the diminishing watering holes. Lack of systematic surveys means that the same individuals or entire flocks may be counted more than once on different days and in different locations, and depending on vagility even geographically distant observations may be pseudoreplicated. Furthermore, these observations of large flocks are largely juveniles, who either die before breeding or never return to their natal area, and may not be representative of the number of breeding pairs in the local area [8].

The Gouldian finch is distributed across the savannah woodlands of the wet-dry tropical regions of Australia, and across a number of major biogeographic boundaries [9,10] including the Ord Arid Intrusion, which has been previously identified as important in maintaining sub-specific variation in the related long-tailed finch [11]. Therefore, we might expect there to be population genetic structure corresponding to these barriers, depending on the species’ ability to disperse. There are conflicting reports about the movement capacity of the Gouldian finch, which may vary according to the season. Early reports suggest migrations within North Queensland in and out of breeding grounds [12,13], More recently, there have been anecdotal reports of birds travelling long distances between localities and outside the breeding range [7]. In a banded population of Gouldian finches studied at Australian Wildlife Conservancy’s Mornington Sanctuary, in the Kimberley, Western Australia, the maximum distance between re-sightings and recaptures was 20 km [14], and radio-tracking suggests birds can move within a 3 km radius within a day [8]. Australian Bird and Bat Banding Scheme records show the average recovery distance for banded birds within and between years is 1 km. Although the typical banding activity in remote areas in Australia tends to be highly focused on a particular area, and most birds are re-trapped in that area, and very rarely re-trapped at other remote sites due to no, or very low banding effort at other sites. At locations where Gouldian finches are regularly banded, the return rates between years are low compared to co-distributed Estrildid finches (1%-17% in the Gouldian finch, 15–60% in long-tailed finches), and show much variation in the total number of individuals in any given year ([8,14,15], S1 Appendix). It is unknown whether low recapture rates represent high mortality, long distance movement patterns or some combination of these.

Previous population genetics analyses on the Gouldian finch has suggested a lack of any genetic structure and high gene-flow, but relatively limited sampling and the genetic markers used have not allowed for robust conclusions [16,17]. The first population study on this species found no significant differences in allele frequency in the Myoglobin intron from samples taken across the geographic range (three sites in the west and a sample of seven birds from Georgetown in Queensland) [16]. However, a single coding locus may have reduced diversity due to selection and does not provide adequate statistical power to draw conclusions about population connectivity. More recently, data from six microsatellite loci and mitochondrial control region sequences from samples collected at two geographically distant localities (one in Western Australia and one in Northern Territory) indicated no evidence of structure [17]. It is possible that structure may not have been detected in this study due to poor geographic coverage of the species’ distribution [18], and the relatively small number, and characteristics of the loci employed may have lacked sufficient power to detect weak differentiation [19]. Additionally, while this study explored historical gene-flow, it is of greater use to management to compare these estimates to those from methods capable of assessing current gene-flow [17]. In this paper, we perform a thorough investigation of Gouldian finch population genetics, to determine whether population structure exists, particularly across the major biogeographic boundary of the Ord Arid Intrusion and across the Kimberley Plateau. We used mitochondrial control region sequences, sixteen microsatellite markers, and 3,839 SNP loci to infer levels of genetic diversity and differentiation across the Gouldian finch range. From these data, we ask whether locations that are reliable for catching and sighting Gouldian finches should be considered separate management units. Finally, we explore these results in the context of diversity in a co-distributedAustralian finch, and the consequences of our results for conservation of the Gouldian finch.

## Methods

### Sample collection

**Fig 1:**
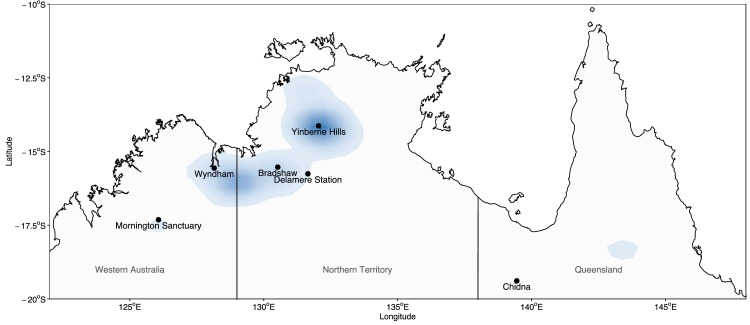
Map of the north of Australia, showing the locations blood samples were collected between 2004 and 2013. Heat map indicates the density of Gouldian finch presence data from Atlas of Living Australia [20] since trapping became illegal in 1987, where darker blue indicates high occurrence density. Background map reprinted from [22] under a CC BY 4.0 license, with permission from the Australian Bureau of Statistics, Original Copyright 2011.

Samples were collected from six locations across the range of the Gouldian finch in Australia between 2004 and 2013 (Fig 1). The sampling localities focus on areas of historical and contemporary high abundance in the Gouldian finch, reflected in the heat-map based on occurrence density in Fig 1 from Atlas of Living Australia data [20]. These data are from 1987 onwards, when Gouldian finch trapping was banned. We refer to these five sites (Mornington through Yinberrie Hills) in this area as “the western range”. Considerably fewer individuals were caught at Chidna because Gouldian finches appear to be less abundant in the east [7]. The majority of birds were caught in the breeding season (late wet season; January-August). Birds at all sites except Wyndham were banded and bled next to waterholes. Birds were caught using a mist-net over a few hours after dawn. Under Australian federal law, all birds must be fit with a unique metal band supplied by the Australian Bird and Bat Banding Scheme (ABBBS), and the handler must be certified by the ABBBS as a competent handler and bander. Blood was drawn by puncturing the brachial vein with a 26-gauge needle and collecting a sample volume of <60μL with a capillary tube. Samples taken for the purposes of genetics and were bled immediately after capture, those taken for hormone analysis were taken between 5 and 60 minutes after capture [21].The birds were restrained using ringers grip, no anaesthetics were administered as the bleeding procedure takes less than two minutes per bird. Blood samples were stored in 95% ethanol or Queen’s Lysis Buffer.

As part of a larger study to compare breeding ecology of two sympatric hollow-nesting finches, the site at Wyndham was supplemented with nest-boxes and the breeding of Gouldian and long-tailed finches was monitored [15,23,24]. Birds were caught at nests with hand nets when nestlings were more than 14 days old to avoid nest desertion. Birds were also caught at nearby waterholes with mist-nets and provided with unique bands and bled as described above. Under both scenarios birds were confined for less than ten minutes. For comparison with long-tail finches at the same site [15], we provide recapture rates for different life-history stages of the Gouldian finch across six years (S1 Appendix).

For different analyses and molecular techniques we used different subsets of samples, based on quality and quantity of DNA extract. For each analysis, we aimed for similar sample sizes across all five extensively sampled populations, because sample size differences can bias some estimates of genetic differentiation [25].

### Molecular methods and analysis of genetic data

Gouldian finch DNA was extracted from blood samples using a Qiagen PureGene Kit, and subsequently used in microsatellite, mtDNA and genotyping-by-sequencing analyses.

#### Microsatellites

Because we had access to a large number of samples over a 10 year period, microsatellite analyses were conducted with two different subsets of individuals to answer different questions about genetic structure. We used 93 breeding individuals from the Wyndham population in 2008 and 2009 [23,24,26] to examine fine-scale genetic structure. All other analyses are based on a random subset of individuals matched to the sample size of the smallest locality (49 individuals), plus an additional six individuals from Chidna in Western Queensland. Twenty-two microsatellite loci were amplified across five multiplexes, according to protocols listed in S2 Appendix. These 22 loci were checked for amplification consistency, null alleles, and conformity to Hardy-Weinberg Equilibrium (S2 Appendix) using ARLEQUIN v.3.5.2.2 [27]. Linkage disequilibrium between loci was tested in GENEPOP v4.2 [28]. Hardy-Weinberg and linkage disequilibrium were corrected for multiple testing using Bonferroni correction. Loci that were inconsistent or violated assumptions were removed from downstream analyses.

We then used ARLEQUIN to tabulate the overall allelic richness (N_A_), observed and expected heterozygosities (H_O_, H_E_), and GenAlex v6.502 to calculate private alleles [29]. Differences in allelic richness and heterozygosity in each of the five major sampling localities was conducted by pairwise Wilcoxon sign-rank tests, and Bonferroni corrected for multiple testing. Measures of allelic richness are sensitive to differences in sample size, so allelic richness per locus at the five major localities was rarefied to match the sample size at Chidna using ADZE 1.0 [30]. The rarefied richness values were then compared using pairwise Wilcoxon sign-rank tests with Bonferroni correction.

To obtain measures of global differentiation and inbreeding, we conducted an Analysis of Molecular Variance (AMOVA) in ARLEQUIN [27] with 10,000 permutations. We calculated the number of private alleles per population using GenAlex [29,31]. We used pairwise F_ST_ and AMOVA (global F_ST_) to estimate genetic structure between sampling localities in ARLEQUIN [27], with 10000 permutations to identify statistical significance. Given that F_ST_ can under-estimate differentiation at highly polymorphic loci [32–34], for all pairwise comparisons we also calculated Jost’s D [35] using R package ‘*DEMEtics*’ with p-values calculated by 1000 bootstrap resamples [33]. Continuous populations may not exhibit evidence of pairwise or global differentiation, but may show a pattern of increased differentiation with distance—isolation by distance (IBD). We measured IBD in ARLEQUIN using a Mantel test against a matrix of pairwise geographic distances between sampling localities, with 10000 permutations [27]. We also conducted individual-based genetic clustering analyses because sampling localities may not reflect actual populations [36]. These techniques are useful because they find genetic clusters without *a priori* population definitions. We used STRUCTURE v 2.3.4 [37] to estimate the number of genetic groups in our dataset. We compared the effect of the location as a prior (LOCPRIOR: [38]) against the standard model with no location prior on the resulting genetic clusters. The location prior does not define populations strictly *a priori*, but considers individuals that are sampled together to be more likely from a genetic cluster [38]. This method is sensitive to subtle population structure, but will not falsely detect structure [38]. We used admixture models with correlated allele frequencies. The length of the burn-in was 100,000, followed by 1,000,000 MCMC, with K (number of clusters) set between 1–10 and 10 iterations per value of K. K was determined by comparison of plots of Ln P(D) and Δ*K* [39] using STRUCTURE HARVESTER v0.6.94 [40].

As an independent assessment of number of genetic clusters, we also ran a population genetic model-free ordination clustering method in R-package ‘*adegenet’* [41]. These ordination techniques have the advantage that it does not rely on any particular population genetic model (such as minimising deviation from Hardy-Weinberg equilibrium as in STRUCTURE) to discern the number of clusters [41,42]. We used the *find*.*clusters* function to select clusters, which reduces the genetic data into Principal Components and runs a k-means clustering analysis (where k is the number of clusters) and weights results according to the Bayesian Information Criterion (BIC), and we retained all principal components for this analysis. Subsequently, we performed a Discriminant Analysis of Principal Components (*dapc*) using R-package ‘*adegenet*’ [41,42], which takes *a priori* clusters and maximises the distances between them. We also ran *dapc* using the collection localities as prior groupings in an effort to explore geographic structuring by maximising the multivariate distances between sampling localities. Using the selected clusters, we ran model validation on ability of the model to correctly assign individuals to their clusters using *optim*.*a*.*score* and *xvalDapc* functions. For more information please see S4 Appendix.

Some movement information we have on Gouldian finches, based on band recoveries and radio-tracking, suggests that many individuals may be restricted to a 5km area, at least over short periods. Low recovery rates in the banding data suggest very limited natal philopatry in these birds, but we used genetic data as an independent test of this. If movement is limited, and natal philopatry is high, we might expect to see some evidence of spatial structure on the scale of a few kilometres. Spatial autocorrelation takes pairwise genetic distances and pairwise geographic distances between individuals and provides a measure of autocorrelation (r)–and by proxy genetic similarity—between them [43]. Under restricted dispersal, geographically proximate individuals should have shorter genetic distances between them, and will show a signature of positive autocorrelation at this spatial scale. We used this spatial autocorrelation approach to investigate restricted movement at the local scale. Spatial autocorrelation was conducted on individuals nesting in 2008 and 2009 over ~12km in our Wyndham study site [24,26]. Significant autocorrelation (either positive or negative) was determined by 1,000 bootstrap resamples against 1,000 permutations of a null hypothesis constituting no spatial structure [43]. All analyses were conducted in conducted in GenAlex v6.502 [29,31], and were partitioned according to sex to investigate whether there was sex-biased dispersal [44].

#### mtDNA

For a subset of individuals (sample sizes in Table 1), we sequenced mitochondrial control region domain 1. We amplified a 330bp segment using primers and protocols developed in the closely related long-tailed finch [11]. Final sequences were checked using python programme SEQTRACE v 0.9.0 [45].

**Table 1:** Summary of various measures of genetic diversity with (±) sampling standard deviation for microsatellite, mitochondrial and SNP datasets.

Parameter	Mornington	Wyndham	Bradshaw	Delamere	Yinberrie Hills	Chidna	Overall
*Microsatellites*							
**N**	49	49	49	49	49	6	251
**N**_**A**_	13.88	14.13	14.31	14.31	14.63	5.69	21.81
**N**_**PA**_	1.06	0.88	1.31	1.06	1.12	0.18	NA
**H**_**O**_	0.77 (±0.12)	0.77 (±0.13)	0.76 (±0.15)	0.79 (±0.14)	0.77(±0.14)	0.80 (±0.15)	0.77 (±0.12)
**H**_**E**_	0.81 (±0.12)	0.81 (±0.11)	0.81 (±0.12)	0.81 (±0.11)	0.81 (±0.11)	0.80 (±0.14)	0.81 (±0.11)
**F**_**IS**_	0.04*	0.04**	0.06***	0.02	0.05**	0.04*	0.04**
*Mitochondrial control region*							
**N**	32	35	25	32	23	5	152
**S**	6	9	10	5	6	4	14
**H**	8	10	12	5	8	3	20
**H**_**R**_	6.8 (± 0.96)	7.8 (± 1.11)	11.4 (± 0.66)	4.7 (± 0.46)	NA	NA	NA
**H**_**P**_	1	2	6	0	2	0	NA
***h***	0.68 (± 0.07)	0.80 (± 0.04)	0.83 (± 0.07)	0.71 (± 0.06)	0.71 (± 0.09)	0.70 (± 0.22)	0.76 (± 0.07)
**π x10**^**2**^	0.37 (± 0.07)	0.50 (± 0.07)	0.59 (± 0.10)	0.39 (± 0.07)	0.51 (± 0.08)	0.61 (± 0.21)	0.47 (± 0.03)
*Genotyping-By-Sequencing SNPs*							
**N**	52	47	48	53	48	3	251
**N**_**S**_	3817.7 (±18.9)	3816.0 (±20.9)	3826.9 (±10.2)	3818.4 (±20.5)	3823.3 (±17.9)	3827.7 (±4.0)	3820.3 (±18.3)
**S**	3838	3836	3837	3835	3837	2497	3839
**H**_**O**_	0.30 (±0.17)	0.30 (± 0.17)	0.30 (±0.17)	0.30 (± 0.17)	0.30 (±0.17)	0.48 (± 0.26)	0.30 (± 0.16)
**H**_**E**_	0.30 (± 0.15)	0.30 (±0.15)	0.30 (± 0.14)	0.30 (± (0.15)	0.30 (± 0.14)	0.47 (± 0.11)	0.30 (± 0.14)

The table describes each population, the number of individuals used in the analysis (N), and the observed heterozygosity (H_O_), expected heterozygosity (H_E_), number polymorphic sites (S). Diversity measures specific to the microsatellites are: the average no alleles per locus (richness) (N_A_), number of private alleles per locus (N_PA_), and Inbreeding Coefficient (F_IS_), with degree of significance indicated by number of asterisk. Diversity measures specific to the mitochondrial data are: raw number of haplotypes (H), rarefied number of haplotypes to n = 23 (H_R_), private haplotypes (H_P_), haplotype diversity (*h*), and nucleotide diversity (π). Measures specific to SNPs are the average number of sites across individuals in a population (N_S_). NA indicates the parameter was not calculated for that population, either due to sample size constraints, or it was not a relevant parameter.

* is p<0.05

** is p<0.005

*** is p<0.0005

We examined the mitochondrial genetic diversity from a subset of individuals across the five western populations, plus we explored the five individuals from Chidna in the east. Haplotype richness (H), haplotype diversity (*h*) and nucleotide diversity (π) was calculated using DnaSP v5.10.1 [46]. We calculated the rarefied haplotype richness (H_R_) based on the smallest sensible sample size (at Yinberrie Hills, n = 23) was calculated in Analytic Rarefaction 2.0 [47].

We conducted an AMOVA and population pairwise genetic differentiation measures in ARLEQUIN. We calculated differentiation between sampling localities using haplotype frequencies (F_ST_) and nucleotide diversity ϕ_ST_ calculated with the Kimura 2-parameter model. All significance tests were run with 10,000 permutations. In addition, we examined the evolutionary relationships between haplotypes using a median-joining network (ε = 0, [48]) using the programme PopART (http://popart.otago.ac.nz).

#### Genotyping-by-sequencing

We also used a reduced-representation next generation sequencing approach to obtain SNPs from across the genome. We sequenced 285 individuals that included a subset of individuals from our six populations (Mornington = 56, Wyndham = 57, Bradshaw = 57, Delamere = 56, Yinberrie Hills = 55, Chidna = 4). Populations were randomised across three plates, and 12 individuals were duplicated across all three plates, to ensure there were no lane or library effects, which can cause artificial substructure in the data.

We sent DNA extracts to Cornell Institute of Genomic Diversity for library preparation and sequencing according to their in-house Genotyping-by-Sequencing (GBS) methodology [49]. This is a reduced representation approach, similar to RADseq [50], that sequences short sections of the genome downstream from restriction enzyme cut-sites. We used restriction enzyme *Pst1*; *in silico* digests using ‘*SimRAD*’ suggested this enzyme yields 952,644 cut-sites across the genome of the related Zebra finch *Taeniopygia guttata* [51,52]. Each plate was multiplexed into three lanes on an Illumina HiSeq 2500 (100bp single-end reads).

Raw reads were processed into SNP genotypes using the reference-free bioinformatics pipeline designed for this particular methodology: Universal Network Enabled Analysis Kit (UNEAK) [53] in TASSEL 3.0 [54]. Reads must have been recorded at least five times to be included as a tag for further analysis, and an error tolerance rate of 0.03 was used to identify reciprocal tag pairs for SNP calling [53]. SNPs with a minimum minor allele frequency (mnMAF) of 0.00, 0.01 and 0.05 were called across the entire dataset using TASSEL. We compared the results of the three mnMAFs, and they did not affect the interpretation so we chose the most conservative dataset (mnMAF = 0.05). All resulting SNPs were subsequently filtered using VCFtools [55] according to the following quality criteria: a) minimum read depth of five reads per genotype; b) sites with an outlying number of reads, as these can represent gene duplications or repetitive regions. An arbitrary threshold of >28X was chosen based on the frequency histogram of the number of reads; c) removing individuals with outlying high and low average sequencing depths, and overall heterozygosity by removing the bottom and top ten percent of individuals in these categories. Then the data were filtered for missingness according to site and individual criteria: a) sites not represented in >80% of individuals [56]; b) individuals with >50% or >30% missing genotypes. Filtering individuals by >30% or >50% missing genotypes did not affect the results, so we present the data based on those with more individuals (>50%).

We measured heterozygosity, number of polymorphic sites per population using ARLEQUIN v.3.5.2.2 [27]. We conducted an AMOVA and pairwise F_ST_ between sampling localities using pairwise distance for the underlying distance matrix in ARLEQUIN [27], and 10,000 permutations.

Genetic clustering analysis was conducted in a Bayesian clustering programme FASTSTRUCTURE v1.0 designed to process large SNP datasets quickly [57]. This programme is based on a variational Bayesian inference framework, which does not necessitate user set sampling of parameter space (e.g. MCMC reps). As a preliminary step to detect strong genetic structure in the SNP data, we ran models with 1 to 10 genetic clusters (K), using a ‘simple’ or flat prior of population specific allele frequencies. This method reports two new measures of K that explain the data in different ways, and can provide a range of K values to run further tests with more sensitive priors [57]. As in the microsatellite dataset, we also ran a discriminant analysis on this data to infer genetic clusters.

### Mitochondrial richness in co-distributed Australian finches

Genetic diversity is reduced in threatened taxa, relative to non-threatened taxa reflecting population bottlenecks and small population sizes [58–61]. We used data from the same section in the mitochondrial control region from a related and co-distributed finches to provide context for the measures of genetic diversity. We compared genetic diversity in the co-distributed, but ‘Least Concern’ status long-tailed finch (*Poephila acuticauda*) using previous data from the mitochondrial control region [11]. Measures of allelic richness are more sensitive to population size changes than diversity or heterozygosity [62], but must be corrected for sample size [63]. We rarefied haplotype richness of the long-tailed finch (n = 274) to match the smaller sample of the Gouldian finch (n = 152) in the program Analytic Rarefaction 2.0 [47]. Point estimates of rarefaction cannot be directly compared statistically, but we used the rarefaction sampling variance and 95% confidence intervals as a guide for whether mitochondrial control region haplotype richness was different between the different species. Estimates of diversity are much less sensitive to sample size, and therefore we directly compared uncorrected haplotype and nucleotide diversity estimates. Providing a sufficiently large sample size, central limit theorem predicts that the nucleotide diversity and haplotype diversity sampling variance derived from theory will approximate a normal distribution [64], which allowed us to use a t-test to compare diversity indices.

## Results

After quality filtering of the microsatellite dataset (S2 Appendix), we retained 16 of 22 loci that were in Hardy-Weinberg Equilibrium (after Bonferroni correction p<0.000625) (Table B in S2 Appendix). No pairs of loci were in linkage disequilibrium after Bonferroni correction (Table C in S2 Appendix).

Summary diversity statistics for the microsatellite data are presented in Table 1. Within collection localities, the microsatellite allelic richness was between 13.8 and 14.6 in the five major populations, and pairwise Wilcoxon sign-rank tests found no significant differences in richness at our five major sampling localities (Bonferroni corrected p-values = 0.36–1). Pairwise tests on rarefied richness to include the smallest sample at Chidna also found no significant difference (all Bonferroni corrected pairwise p-values = 1). Similarly, there was no significant difference in observed or expected heterozygosity between any of the localities (all Bonferroni corrected pairwise p-values = 1). For the uncorrected pairwise p-values please see Tables A and B in S3 Appendix.

The sample sizes for populations using mtDNA were smaller than those employed in the microsatellite analysis. Of the 330bp fragment amplified, 14bp were polymorphic and we identified 20 haplotypes (KX858950-KX585969). There was considerable variation in levels of mitochondrial richness between the collection localities (Table 1). Bradshaw locality had the highest private haplotype count and contained 60% of the total observed haplotypes, and had higher haplotype richness than the site with next highest richness (Wyndham rarefied to n = 25, H_R_ = 8.2, 6.14–10.0 95% CI, Bradshaw raw H = 12). The haplotype diversity was not significantly higher (t_1.93_, df = 35.15,p = 0.062), but nucleotide diversity was significantly higher in Bradshaw than Wyndham (t_3.87_, df = 40.26, p = 0.00039).

Genotyping-by-sequencing (GBS) yielded 735,164,326 raw reads across three Illumina lanes containing 96 samples each, with an average of 2,552,653.91 reads per individual or 1.6X reads per predicted site. After stringent filtering, we retained 3839 SNPs with a minimum site depth of 5X and average 13.5X across 251 individuals. Like the microsatellites, measures of genetic diversity were consistent across the five main sampling localities (Table 1). No sampling localities had private alleles, but this is expected due to the high minimum minor allele frequency filtering.

### Population structure

Analysis of molecular variance (AMOVA) on the microsatellite data showed that most genetic variation was contained within individuals, with less than 1% of variation attributed to between population differences, and 4.2% among individuals within populations. Inbreeding coefficients (F_IS_ and F_IT_) were low but statistically significant (F_IS_ = 0.042, p = 0.00; F_IT_ = 0.043, p = 0.00) based on permutation tests, and individual population inbreeding coefficients (F_IS_) are not consistent across populations (Table 1). SNP results showed similarly low variation between populations, but there was no indication of inbreeding (F_IS_) using these markers (F_ST_ = -0.0001,p = 1; F_IT_ = -0.093, p = 1; F_IS_ = -0.093, p = 1). AMOVA based on mtDNA nucleotide diversity and haplotype frequency yielded slightly higher between population differentiation, but still with only 1.9% of genetic variation explained among sampling localities.

**Fig 2:**
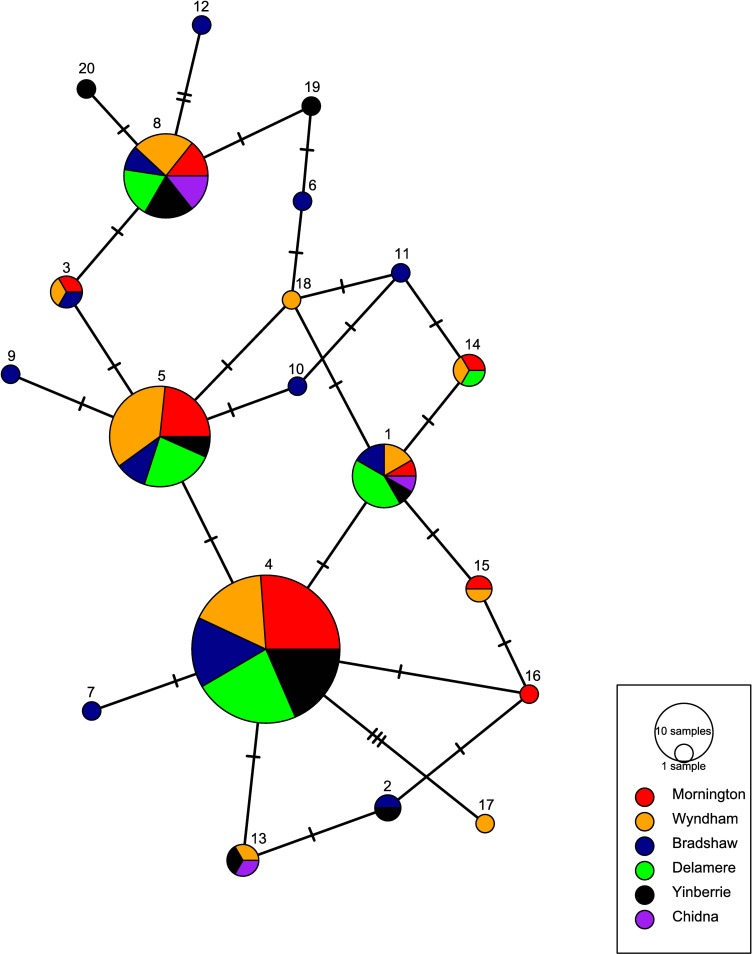
Median-joining network for mitochondrial control region haplotypes in the Gouldian finch. Colours represent sampling localities, and node circle size represents the number of individuals with that haplotype. Number of strokes joining nodes indicates then number of mutations between two haplotypes.

All measures of pairwise genetic differentiation between the collection localities was negligible and statistically non-significant after Bonferroni correction (Tables C-E in S3 Appendix). Haplotype frequencies between Chidna and all other populations were moderately differentiated (average Chidna Pairwise F_ST_ = 0.20, standard deviation = 0.046), and nucleotide diversity was significantly differentiated (average ϕ_ST_ = 0.25, standard deviation = 0.085) except with closest neighbour Yinberrie Hills, but none of these comparisons were significant after Bonferroni correction (Table D in S3 Appendix). The lack of population structure at mtDNA can be visualised in the median-joining network (Fig 2), which shows no pattern of unique haplotypes shared between different regions.

Mantel tests showed evidence of Isolation-by-Distance (IBD) in the mitochondrial dataset (ϕ_ST_, β = 0.00034, p = 0.026; F_ST_ β = 0.00027, p = 0.039), and a significant effect in the microsatellite dataset (β = 9x10^-6^, p = 0.0064) and SNP datasets (β = 1x10^-5^, p = 0.04), but the regression coefficients in both analyses was small. When the poorly sampled and distant Chidna locality was removed from the analysis there was no significant effect in the microsatellite dataset (β = 0, p = 0.53), SNP dataset (β = 0, p = 0.43), or mitochondrial dataset (ϕ_ST_, β = 0.000021, p = 0.27; F_ST_ β = -0.000004, p = 0.43).

**Fig 3:**
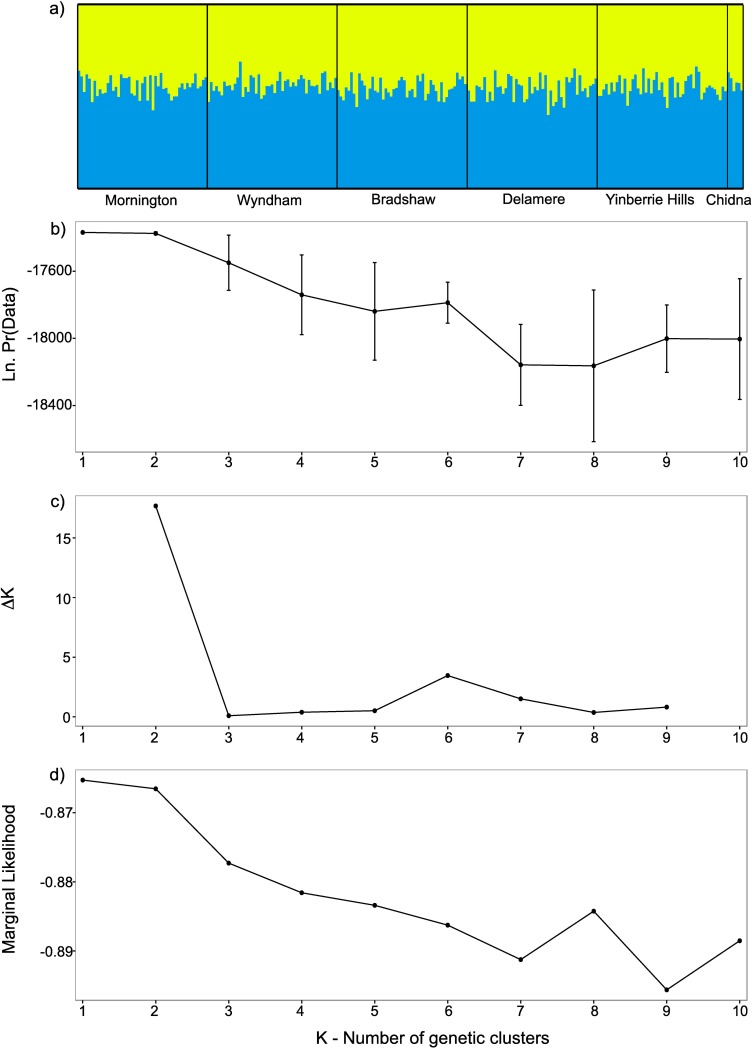
**Results from Bayesian clustering analysis using STRUCTURE (a-c)** [37] **and d) fastSTRUCTURE** [57]. Part a) shows equal membership probability plot for each individual plotted for two clusters; b) log probability of data (LnP(D)) showing K = 1; c) the optimal number of genetic clusters according to the Evanno et al method; d) output of marginal likelihoods from fastSTRUCTURE showing optimal K = 1.

There was no evidence of spatial genetic clustering from the STRUCTURE analysis. The results did not differ meaningfully with or without the use of the LOCPRIOR model [38], so we only present the latter results here. In the LOCPRIOR model, if the parameter *r* is less than one, it suggests that the location information is informative to the ancestry of the location [38]. Across all our repetitions, the mean r inferred was well above 1 (mean r = 11.94, standard deviation r = 1.26). The log probability of the data (genotypes given K clusters, LnP(D)) indicates the best model fit is for a single cluster (Fig 3C). If the rate of change in LnP(D) is used to infer number of clusters [39], then we find that the optimal number of clusters (**Δ**K) is two (Fig 3B), but this method is only able to make inferences about clusters greater than or equal to two. Indeed, the LnP(D) plot only shows a strong drop off in model fit after K = 2. Therefore, we plotted the ancestry proportions for each individual given two clusters. All individuals are equally admixed (Fig 3A) across the range, supporting a single genetic cluster. Furthermore, the fastSTRUCTURE method on the SNP dataset found the optimal number of clusters was one at both measures of K (Fig 3D).

**Fig 4:**
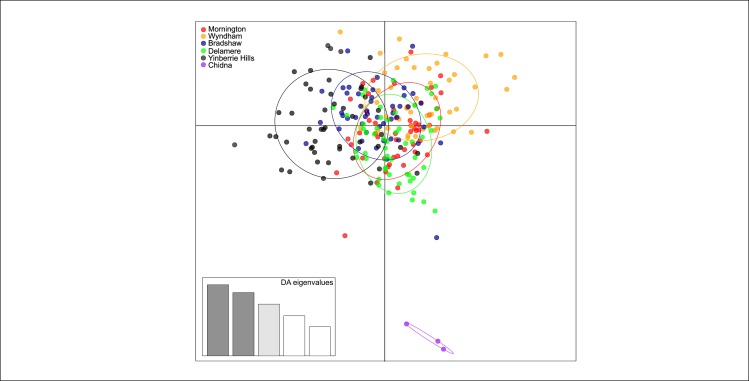
Scatterplot from discriminant analysis of principal components on the Genotyping-by-Sequencing SNP dataset. Points represent individual genotypes, and colours are the sampling localities surrounded by a 95% confidence ellipse. DA eigenvalues represent the amount of genetic variation captured by the discriminant factors plotted as the x- and y- axis.

In the microsatellite and SNP dataset, the k-means clustering method *find*.*clusters* in ‘adegenet’[41] found that the lowest BIC was for one cluster (K = 1)(Figs A and D in S4 Appendix). When running Discriminant Analysis of Principal Components (DAPC) using collection locality as the grouping factor, we found no evidence of separation between all six localities in the microsatellite dataset. Yet, in the SNP data DAPC was able to distinguish the three samples from Chidna (Fig 4). For more details on the procedures and model validation see the S4 Appendix.

The spatial autocorrelation also revealed no pattern of fine scale genetic structure (Fig A in S3 Appendix). When the sexes were considered separately in the spatial autocorrelation there was also no evidence of sex-bias in spatial genetic structure patterns (results not shown).

### Mitochondrial richness in co-distributed Australian finches

The haplotype richness observed in the Gouldian finch fell within the confidence intervals of the rarefied long-tailed finch estimate (n = 152, H_R_ = 21.2, 17.19–25.23% CI). Conversely, haplotype diversity and nucleotide diversity were significantly higher in the long-tailed finch (*h*, t_-9.04_, df = 321.68, p<2.2x10^-16^; π, t_-24.01_, df = 212.64, p<2.2x10^-16^).

## Discussion

Genetic diversity is an important indicator of future adaptive potential and risk of inbreeding [65]. In the Gouldian finch, there was no evidence in SNP and microsatellite markers for differences in genetic diversity between populations, but the population at Bradshaw had higher mitochondrial richness and diversity than the other populations. This is not the result of a confluence between haplotypes that are abundant or private to localities to the east and west, as most haplotypes are found throughout the range, and appears to be driven mostly by a higher number of private haplotypes. This pattern in allelic richness may represent a gradient of genetic diversity between range core (Bradshaw) and toward the range edge populations (See Fig 1 observations from Atlas of Living Australia) [20,66]. Across the sampled range, mitochondrial haplotype richness does not appear to be different between the Gouldian finch and related and ‘Least Concern’ long-tailed finch (*Poephila acuticauda*), but mitochondrial diversity estimates are significantly lower in the Gouldian finch than the long-tailed finch (*P*. *acuticauda*). Indeed, the widespread zebra-finch (*Taeniopygia guttata*) has similarly high estimates of diversity at mtDNA locus ND2 as those observed the long-tailed finch [11,67]. Broadly, the lower haplotype diversity in Gouldian finches supports a “Threatened” conservation status. However, measures of nucleotide diversity do not correlate well with population size or bottleneck intensity [68–70], therefore lineage specific processes (such as mutation rate or life-history) might be more important in determining mitochondrial diversity between these two species. Therefore, we caution against conclusions about the population status of the Gouldian finch until formal analyses of effective population size have been conducted.

We found evidence of heterozygote reduction (F_IS_ and F_IT_ = 0.04) in the microsatellite dataset, but not the SNP dataset. This discrepancy is potentially because of large sampling variances associated with these highly polymorphic markers, in conjunction with our modest number of markers and population sampled, and therefore expected heterozygosities from SNP data may be more accurate [71,72]. Further, small microsatellite marker sets are not good predictors of pedigree inbreeding except in highly structured populations with high levels of consanguineous matings, and therefore may not correlate well with inbreeding depression ([72,73] but see [74]). That we find no evidence of increased F_IS_ & F_IT_ inbreeding coefficients in the more representative and statistically powerful SNP dataset suggests that inbreeding may not occur at significant levels in the wild Gouldian finch. Indeed, field observations suggest consanguineous matings may be rare, due to very low numbers of recaptured individuals between years, suggesting high levels of dispersal ([8,14], S1 Appendix). Further, although banding data indicate that individual movements occur over small spatial scales, our spatial autocorrelation data indicate that relatedness is not spatially structured within the Wyndham sampling site. Together, the genetics and banding data suggest that birds become more mobile after the breeding season, or that adult mortality is high and juveniles are highly dispersive.

We found no evidence of genetic differentiation in the western range of the Gouldian finch from Mornington to Yinberrie Hills, a distance of 730 km, despite the bio-geographic complexity and vast distances involved in this part of Australia [10]. Indeed, we find no fine-scale genetic structure at the landscape scale in Wyndham, nor evidence of Isolation-by-Distance across the five major sampling localities (spanning 730km) across our three marker types. Notably, our sampling spans a number of biogeographic breaks including the Ord Arid Intrusion (Wyndham area) and the Victoria Gap, which have been associated with genetic discontinuities within a number of species with different dispersal capacities [10,75,76]. However, we found no evidence of a genetic discontinuity in the Gouldian finch across these barriers. The Ord Arid Intrusion is associated with separate mtDNA lineages roughly in line with the sub-species of the long-tailed finch (*P*. *a*. *acuticauda* and *hecki*) [11]. This is congruent with our understanding of movement based on mark recapture studies of both species, where long-tailed finches show more site-fidelity than the Gouldian finch ([8,15], S1 Appendix). Further, no genetic structure has been previously detected in the nomadic continental zebra finch, and therefore it is plausible that Gouldian finches are similarly dispersive [77], but equally plausible is an expansion from a single ice-age refugium as seen in many Palaearctic and Nearctic faunas [78].

Genetic differentiation between the western range and our sampling locality in Queensland, Chidna, remains unclear. The Chidna locality was responsible for significant results in Mantel tests for IBD, despite Chidna showing no evidence of significant pairwise differentiation after Bonferroni correction. Discriminant Analysis of Principal Components on the SNP dataset identified Chidna as a distinct cluster, but our validations suggest model instability. However, clustering analyses found no evidence that there was more than one genetic cluster, suggesting Chidna is part of the same population. Because of the small sample size at Chidna it is unlikely that we have captured the true allele frequencies at the location, and any signal of differentiation (or lack-thereof) may be spurious [25,71]. However, anecdotal reports suggest Gouldian finch densities are lower in Queensland [7], which differs in land-management and fire-regimes which are determinants of Gouldian finch abundance [5,14,79,80]. These tracts of unsuitable or poor quality habitat may restrict movement between the western range and Queensland, and within Queensland, which may be severe enough to restrict gene-flow. Therefore, we urge that results of high genetic connectivity in the western range are not extrapolated into Queensland.

The putative decline and fragmentation of the Gouldian finch (the 1960s-1970s) is relatively recent (30–50 years and Gouldian finch generations), and there may not have been sufficient time to detect a reduction in gene-flow [78,81]. The time it takes for a reduction in gene-flow to affect allele frequencies will depend on the migration rate, effective population size of the subpopulations, generation time and overlap and population growth [82,83]. However, modelling has shown a complete cessation of gene-flow can be statistically detectable within two generations using equilibrium estimators (e.g. F_ST_) for microsatellite sampling schemes equivalent to ours and census population sizes of less than 500 individuals [19]. To our knowledge, there is no equivalent modelling done on high-throughput technology SNP data, but studies suggest that the number of SNPs employed here ought to be sufficient to distinguish very low levels of differentiation (e.g F_ST_ <0.05) [19,84–86]. Compared with previous attempts to measure population structure in the Gouldian finch [16,17], we are confident of our finding of the absence of genetic structure across the western range of the Gouldian finch because we included more sampling localities, analyses with different underlying assumptions, and more powerful genetic markers (SNPs) for detecting subtle differentiation [1,19,84,86].

Populations across the western range of the Gouldian finch are genetically interconnected, and exchange more than sufficient effective migrants to maintain the genetic diversity in each region (irrespective of stringency of N_e_*m*) [1,87,88]. But this does not usefully inform the demographic connectivity between regions, as this depends on the subpopulation size and the migration rate between them (*m<*0.1) [1,89,90]. Unless there is detectable differentiation between subpopulations, assignment-test methods such as BayesAss will not be able to measure migration rate between populations [91]. Therefore, ecological data is still useful to infer management units and demographic connectivity between populations [89]. Banding data from Mornington, Wyndham, Newry and Yinberrie Hills indicate very low return rates between years at collection localities ([8,14], S1 Appendix), which suggests that local recruitment (on the scale of the sampling localities) may not be important for maintaining populations. Limited spatial and temporal banding data do not allow inference about Gouldian finch populations beyond what is possible with the genetic data. Until there is an extremely substantial banding effort (both spatially and temporally), or satellite telemetry is used to monitor Gouldian populations across the range (for which tags are currently not available due to the size of the bird), it will remain uncertain whether Gouldian finches are regularly dispersing long distances, or the lack of population genetic structure comes from a high volume of local migrant exchange [3].

All three molecular markers employed in this study provided congruent evidence about moderately high genetic diversity across the western range of the Gouldian finch, with no evidence of genetic differentiation despite biogeographic barriers. Although these data make it impossible to infer demographic connectivity (migration rate, *m*) between populations, we urge caution in the interpretation of spatially and temporally unsystematic estimates of population from anecdotal reports by bird-watchers. Our findings do not exclude the possibility that individual Gouldian finches may be capable of moving quite long distances. The genetic connectivity between the west and the populations in Queensland remain unresolved, but due to differences in land management practices and Gouldian finch density, movement patterns may be drastically different from what we have observed in the western range. Establishing patterns of genetic connectivity in Queensland remains a priority for adequately assessing the population status of the Gouldian finch in Queensland.

## Supporting Information

S1 Appendix Gouldian finches at Wyndham. Detailed description of methods and recapture rates of Gouldian finches at Wyndham between 2008 and 2013.
(DOCX)

S2 Appendix Microsatellite methods. Detailed methods for microsatellite amplification and quality checks.
(DOCX)

S3 Appendix Pairwise diversity, differentiation and spatial autocorrelation results. Figures and tables include uncorrected p-values for differences between each population comparison, pairwise estimates of genetic differentiation and spatial autocorrelation.
(DOCX)

S4 Appendix K-means clustering and discriminant analysis of principal components. Text and figures describe the detailed methods and model checks associated with the analysis of the microsatellite and SNP data in adegenet.
(DOCX)
