# Supplementary material for: Three Molecular Markers Show No Evidence of Population Genetic Structure in the Gouldian Finch (Erythrura gouldiae)
Source: PLoS One. 2016 Dec 9;11(12):e0167723. doi: 10.1371/journal.pone.0167723 (PMC5147959; doi:10.1371/journal.pone.0167723)
Supplement: S1 Appendix — Detailed description of methods and recapture rates of Gouldian finches at Wyndham between 2008 and 2013. (DOCX) [file pone.0167723.s001.docx]

**S1 Appendix: Gouldian finches at Wyndham**

Previous banding efforts at Yinberrie Hills and Newry showed that there was much variation in the number of banded Gouldian individuals in a given year, and generally low (but variable) recapture rates (Woinarski & Tidemann 1992). Indeed, in the sample of banded birds from Mornington Sanctuary recovery rates were very low, between 5 and 19% (Legge *et al.* 2015). In all banded birds in our sample from Bradshaw, Delamere and Yinberie Hills, in the Northern Territory, there was only a single recapture at Bradshaw. Below we present a summary of the banded birds and their recapture rates at the Wyndham site in the Eastern Kimberley (Table A). During these years birds were mist-netted at waterholes and nests were monitored at natural cavities and artificial nest-boxes (Brazill-Boast *et al.* 2010, 2013). The longest duration between recaptures was three years, where band number 87233 was banded in 2010, and recovered again in 2013. One individual, 87237 was captured in 2011, 2012 and 2013. The bird recaptured in 2012 was banded in the nest in 2010.

**Table A:** Summary of birds banded at Wyndham, Western Australia. Banded breeding pairs include only nests where both parents were uniquely identified by band number. Nestlings are chicks that were banded at 14 days in the nest, and fledglings were independent young banded with mist nets at water holes.

| Year | Total Adults | Banded breeding pairs | Pairs from previous year | Adults from previous year | Nestlings banded | Nestlings from previous year | Fledglings banded | Fledglings from previous year |
| --- | --- | --- | --- | --- | --- | --- | --- | --- |
| 2008 | 200 | 34 | - | - | 130 | - | 55 | - |
| 2009 | 64 | 23 | 0 | 4 (2.0%) | 125 | 4 (3%) | 11 | 0 |
| 2010 | 20 | 9 | 0 | 2 (3.1%) | 48 | 0 | 0 | 0 |
| 2011 | 22 | 4 | 0 | 1 (5.0%) | 20 | 0 | 47 | 0 |
| 2012 | 35 | 6 | 0 | 3 (13.6%) + 1§ | 19 | 1§ (5%) | 71 | 0 |
| 2013 | 26 | 10 | 1 | 3 (8.6%) + 1§ | 49 | 0 | 2 | 1(1.4%) |

§ Indicates an individual that was re-caught later than one year after its capture, but had no recaptures in the year in between

**References:**

Brazill-Boast J, Griffith SC, Pryke SR (2013) Morph-dependent resource acquisition and fitness in a polymorphic bird. *Evolutionary Ecology*, **27**, 1189–1198.

Brazill-Boast J, Pryke SR, Griffith SC (2010) Nest-site utilisation and niche overlap in two sympatric, cavity-nesting finches. *Emu*, **110**, 170–177.

Legge S, Garnett S, Maute K *et al.* (2015) A Landscape-Scale, Applied Fire Management Experiment Promotes Recovery of a Population of the Threatened Gouldian Finch, Erythrura gouldiae, in Australia’s Tropical Savannas. *Plos One*, **10**, e0137997.

Woinarski JCZ, Tidemann S (1992) Survivorship and some population parameters for the endangered Gouldian Finch Erythrura gouldiae and two other finch species at two Sites in tropical northern Australia. *Emu*, **92**, 33–38.
