# Supplementary material for: Three Molecular Markers Show No Evidence of Population Genetic Structure in the Gouldian Finch (Erythrura gouldiae)
Source: PLoS One. 2016 Dec 9;11(12):e0167723. doi: 10.1371/journal.pone.0167723 (PMC5147959; doi:10.1371/journal.pone.0167723)
Supplement: S2 Appendix — Detailed methods for microsatellite amplification and quality checks. (DOCX) [file pone.0167723.s002.docx]

**S2 Appendix: Microsatellite methods**

We amplified 22 microsatellite loci across five different multiplexes (Table S2.1), including the first use of primers developed in the Gouldian finch (Kim *et al.* 2015). Multiplexes were amplified using a Qiagen multiplex mix in 5uL reactions.

The step-down thermal cycler protocol for multiplex 1,3,4 and 5 was as follows: 95°C for 15 min, 94°C for 45s denaturation, 70°C, 66°C, 62°C, 62°C, 58°C, 54°C annealing temperatures for 1 minute, 72°C for 1 min extension. These cycles were repeated 8 times per annealing temperature. Final extension was 72°C for 10 min.

The step-down thermal cycler protocol for multiplex 2 was as follows: 95°C for 15 min, 94°C for 30s denaturation, 64°C, 60°C, 56°C, 53°C, 50°C annealing temperatures for 90s, 72°C for 90s. These cycles were repeated 10 times per annealing temperature. Final extension was 72°C for 10 min.

Samples were run at Macrogen Inc. on an ABI 3730 machine using a GS-500 LIZ size standard. Genotypes were scored using GeneMapper 3.7 (Applied Biosystems, Foster City, CA, U.S.A.).

Loci Tgu7 and Tgu3 were excluded because they did not amplify consistently within individuals and between populations. Loci were checked for null alleles using CERVUS 3.0.6 (Kalinowski *et al.* 2007), and deviation from Hardy-Weinberg equilibrium using ARLEQUIN 3.5.2.2 (Excoffier & Lischer 2010). We did not include samples from Chidna in these analyses because of the small sample size (N=6). Any locus that had high rates of null-alleles (above 10%) and/or deviated from Hardy-Weinberg in more than two populations were excluded from the further analyses (those marked with an asterisk in Table A). Significant deviation from Hardy-Weinberg was assessed after Bonferroni correction for multiple comparisons at an alpha-level of 0.05 (results not shown).

For the final dataset of 16 loci we present the summary diversity statistics and null alleles as calculated in ARLEQUIN for 49 individuals from each locality (and six from Chidna) in Table B. No locus combination was significantly in linkage disequilibrium after Bonferroni correction (Table C).

**Table A:** Micosatellite primers used for the different multiplexes employed in this paper.

| **Marker** | **Dye** | **Multiplex** | **uM** | **Reference** |
| --- | --- | --- | --- | --- |
| Pco2 | 6FAM | 1 | 0.142 | (Saito *et al.* 2001) |
| Pca7 | VIC | 1 | 0.532 | (Dawson *et al.* 2000) |
| Cuu4 | NED | 1 | 0.212 | (Gibbs *et al.* 1998) |
| Tgu7* | NED | 1 | 0.638 | (Forstmeier *et al.* 2007) |
| Ind41 | 6FAM | 1 | 0.638 | (Sefc *et al.* 2001) |
| Titgata2 | PET | 2 | 0.334 | (Wang *et al.* 2005) |
| Ind28 | 6FAM | 2 | 0.234 | (Sefc *et al.* 2001) |
| Tgu11 | VIC | 3 | 0.1 | (Forstmeier *et al.* 2007) |
| Ind37 | VIC | 3 | 0.8 | (Sefc *et al.* 2001) |
| BF18 | 6FAM | 3 | 0.2 | (Yodogawa *et al.* 2003) |
| Ase24 | 6FAM | 3 | 0.334 | (Richardson *et al.* 2000) |
| Tgu3* | NED | 3 | 0.4 | (Forstmeier *et al.* 2007) |
| Ego26 | 6FAM | 4 | 0.2 | (Kim *et al.* 2015) |
| Ego27* | VIC | 4 | 0.2 | (Kim *et al.* 2015) |
| Ego15 | NED | 4 | 0.4 | (Kim *et al.* 2015) |
| Ego29* | PET | 4 | 0.2 | (Kim *et al.* 2015) |
| Ego49 | 6FAM | 5 | 0.2 | (Kim *et al.* 2015) |
| Ego31* | VIC | 5 | 0.4 | (Kim *et al.* 2015) |
| Ego34 | NED | 5 | 0.2 | (Kim *et al.* 2015) |
| Ego52 | PET | 5 | 0.2 | (Kim *et al.* 2015) |
| Ego45* | PET | 5 | 0.2 | (Kim *et al.* 2015) |

**Table B**: Summary statistics for each microsatellite locus for each population, describing the number of alleles (N_A_), Observed Heterozygosity (H_O_), Expected Heterozygosity (H_E_), Deviation from Hardy-Weinberg by exact test (P) where bolded values are significant at p<0.05, but none are significant after Bonferroni correction (p<0.000625).

| **Population** | **Locus** | **N_A_** | **H_O_** | **H_E_** | **P** |
| --- | --- | --- | --- | --- | --- |
| Mornington | Ego15 | 22 | 0.896 | 0.938 | 0.5814 |
|  | Ego26 | 11 | 0.796 | 0.858 | 0.0541 |
|  | Ego31 | 13 | 0.776 | 0.817 | 0.1501 |
|  | Ego34 | 12 | 0.771 | 0.801 | 0.3241 |
|  | Ego49 | 35 | 0.918 | 0.953 | 0.7182 |
|  | Ego52 | 9 | 0.837 | 0.786 | 0.9749 |
|  | Ase24 | 6 | 0.551 | 0.653 | 0.2309 |
|  | BF18 | 12 | 0.673 | 0.813 | 0.2066 |
|  | Ind37 | 16 | 0.830 | 0.874 | 0.3751 |
|  | Tgu11 | 6 | 0.653 | 0.645 | 0.0046 |
|  | Cuu4 | 14 | 0.787 | 0.869 | 0.1993 |
|  | Ind28 | 7 | 0.667 | 0.761 | 0.0523 |
|  | Ind41 | 26 | 0.915 | 0.945 | 0.3365 |
|  | Pca7 | 18 | 0.918 | 0.927 | 0.2336 |
|  | Pco2 | 3 | 0.571 | 0.535 | 0.0273 |
|  | Titgata02 | 12 | 0.750 | 0.824 | 0.0956 |
| Wyndham | Ego15 | 19 | 0.659 | 0.850 | **0.0105** |
|  | Ego26 | 14 | 0.898 | 0.888 | 0.4876 |
|  | Ego31 | 13 | 0.776 | 0.833 | 0.2006 |
|  | Ego34 | 13 | 0.681 | 0.828 | **0.0050** |
|  | Ego49 | 35 | 0.918 | 0.950 | 0.0606 |
|  | Ego52 | 8 | 0.776 | 0.769 | 0.6567 |
|  | Ase24 | 7 | 0.653 | 0.691 | 0.4118 |
|  | BF18 | 11 | 0.673 | 0.755 | **0.0226** |
|  | Ind37 | 16 | 0.896 | 0.913 | 0.5997 |
|  | Tgu11 | 5 | 0.633 | 0.602 | 0.4427 |
|  | Cuu4 | 12 | 0.771 | 0.867 | 0.1219 |
|  | Ind28 | 8 | 0.688 | 0.736 | 0.3697 |
|  | Ind41 | 26 | 0.878 | 0.946 | 0.1844 |
|  | Pca7 | 21 | 0.959 | 0.926 | 0.5387 |
|  | Pco2 | 4 | 0.510 | 0.592 | 0.2551 |
|  | Titgata02 | 14 | 0.918 | 0.856 | 0.3161 |
| Bradshaw | Ego15 | 19 | 0.878 | 0.911 | 0.2241 |
|  | Ego26 | 12 | 0.939 | 0.880 | 0.8809 |
|  | Ego31 | 13 | 0.837 | 0.861 | 0.1645 |
|  | Ego34 | 17 | 0.755 | 0.831 | 0.7037 |
|  | Ego49 | 37 | 0.959 | 0.953 | 0.0938 |
|  | Ego52 | 10 | 0.755 | 0.800 | 0.1454 |
|  | Ase24 | 6 | 0.469 | 0.610 | 0.1165 |
|  | BF18 | 11 | 0.592 | 0.761 | 0.0281 |
|  | Ind37 | 16 | 0.857 | 0.914 | 0.6709 |
|  | Tgu11 | 7 | 0.551 | 0.610 | 0.2427 |
|  | Cuu4 | 12 | 0.673 | 0.835 | 0.0737 |
|  | Ind28 | 8 | 0.714 | 0.758 | 0.8325 |
|  | Ind41 | 27 | 0.878 | 0.941 | 0.6016 |
|  | Pca7 | 18 | 0.878 | 0.908 | 0.1263 |
|  | Pco2 | 3 | 0.592 | 0.577 | 1.0000 |
|  | Titgata02 | 13 | 0.898 | 0.858 | 0.9113 |
| Delamere | Ego15 | 19 | 0.913 | 0.901 | 0.7962 |
|  | Ego26 | 14 | 0.918 | 0.861 | 0.8284 |
|  | Ego31 | 10 | 0.708 | 0.771 | 0.0963 |
|  | Ego34 | 14 | 0.898 | 0.840 | 0.4814 |
|  | Ego49 | 37 | 0.918 | 0.954 | 0.1532 |
|  | Ego52 | 9 | 0.633 | 0.750 | **0.0268** |
|  | Ase24 | 6 | 0.551 | 0.632 | 0.3308 |
|  | BF18 | 12 | 0.694 | 0.822 | **0.0040** |
|  | Ind37 | 16 | 0.878 | 0.882 | 0.5994 |
|  | Tgu11 | 7 | 0.653 | 0.622 | 0.8376 |
|  | Cuu4 | 11 | 0.898 | 0.856 | 0.9064 |
|  | Ind28 | 8 | 0.714 | 0.757 | 0.0572 |
|  | Ind41 | 25 | 0.837 | 0.942 | 0.4988 |
|  | Pca7 | 24 | 0.959 | 0.944 | 0.4063 |
|  | Pco2 | 4 | 0.551 | 0.611 | 0.6738 |
|  | Titgata02 | 13 | 0.918 | 0.868 | 0.5757 |
| Yinberrie Hills | Ego15 | 22 | 0.837 | 0.878 | 0.1233 |
|  | Ego26 | 12 | 0.939 | 0.877 | 0.1203 |
|  | Ego31 | 14 | 0.776 | 0.848 | **0.0238** |
|  | Ego34 | 14 | 0.735 | 0.792 | 0.1145 |
|  | Ego49 | 39 | 0.959 | 0.941 | 0.9934 |
|  | Ego52 | 8 | 0.673 | 0.783 | 0.0117 |
|  | Ase24 | 7 | 0.571 | 0.644 | 0.3590 |
|  | BF18 | 12 | 0.612 | 0.752 | 0.1000 |
|  | Ind37 | 16 | 0.837 | 0.904 | 0.2669 |
|  | Tgu11 | 8 | 0.653 | 0.643 | 0.5655 |
|  | Cuu4 | 14 | 0.776 | 0.876 | 0.0590 |
|  | Ind28 | 6 | 0.714 | 0.737 | 0.4206 |
|  | Ind41 | 26 | 0.959 | 0.953 | 0.5406 |
|  | Pca7 | 20 | 0.918 | 0.925 | 0.1121 |
|  | Pco2 | 4 | 0.551 | 0.597 | 0.1525 |
|  | Titgata02 | 12 | 0.837 | 0.856 | 0.4371 |
| Chidna | Ego15 | 7 | 1.000 | 0.933 | 1.0000 |
|  | Ego26 | 5 | 0.833 | 0.833 | 0.9448 |
|  | Ego31 | 3 | 0.667 | 0.621 | 1.0000 |
|  | Ego34 | 3 | 0.667 | 0.712 | 1.0000 |
|  | Ego49 | 9 | 1.000 | 0.955 | 1.0000 |
|  | Ego52 | 4 | 0.833 | 0.682 | 0.7919 |
|  | Ase24 | 4 | 0.800 | 0.733 | 1.0000 |
|  | BF18 | 5 | 0.667 | 0.803 | 0.3035 |
|  | Ind37 | 6 | 0.800 | 0.889 | 0.6172 |
|  | Tgu11 | 4 | 0.500 | 0.455 | 1.0000 |
|  | Cuu4 | 7 | 1.000 | 0.911 | 1.0000 |
|  | Ind28 | 5 | 0.667 | 0.742 | 0.5176 |
|  | Ind41 | 8 | 0.667 | 0.939 | 0.0817 |
|  | Pca7 | 8 | 0.833 | 0.924 | 0.4955 |
|  | Pco2 | 4 | 0.833 | 0.712 | 0.7921 |
|  | Titgata02 | 9 | 1.000 | 0.955 | 1.0000 |

**Table C:** Results from linkage disequilibrium analysis conducted in GenePop, bolded p-values are those significant at p<0.05, but no comparisons were significant after Bonferroni correction for multiple testing (p<0.0004132).

| Locus1 | Locus2 | P-Value |
| --- | --- | --- |
| Ego15 | Ego26 | 0.845067 |
| Ego15 | Ego31 | 0.668469 |
| Ego26 | Ego31 | 0.106509 |
| Ego15 | Ego34 | 0.694017 |
| Ego26 | Ego34 | **0.02957** |
| Ego31 | Ego34 | 0.9421 |
| Ego15 | Ego49 | 0.192741 |
| Ego26 | Ego49 | 0.22047 |
| Ego31 | Ego49 | 0.073453 |
| Ego34 | Ego49 | 0.686806 |
| Ego15 | Ego52 | 0.48442 |
| Ego26 | Ego52 | 0.696252 |
| Ego31 | Ego52 | 0.627829 |
| Ego34 | Ego52 | 0.240781 |
| Ego49 | Ego52 | 0.334443 |
| Ego15 | Ase24 | 0.899588 |
| Ego26 | Ase24 | 0.85959 |
| Ego31 | Ase24 | **0.002716** |
| Ego34 | Ase24 | 0.156194 |
| Ego49 | Ase24 | 0.552941 |
| Ego52 | Ase24 | 0.831776 |
| Ego15 | BF18 | 0.360148 |
| Ego26 | BF18 | 0.181914 |
| Ego31 | BF18 | 0.260903 |
| Ego34 | BF18 | 0.1785 |
| Ego49 | BF18 | 0.110341 |
| Ego52 | BF18 | 0.373995 |
| Ase24 | BF18 | 0.06989 |
| Ego15 | Ind37 | 0.13613 |
| Ego15 | Ind37 | 0.13613 |
| Ego26 | Ind37 | 0.305382 |
| Ego31 | Ind37 | 0.744387 |
| Ego34 | Ind37 | 0.716983 |
| Ego49 | Ind37 | 0.585006 |
| Ego52 | Ind37 | 0.700925 |
| Ase24 | Ind37 | 0.955974 |
| BF18 | Ind37 | 0.430907 |
| Ego15 | Tgu11 | 0.489194 |
| Ego26 | Tgu11 | **0.006512** |
| Ego31 | Tgu11 | 0.835162 |
| Ego34 | Tgu11 | 0.065263 |
| Ego49 | Tgu11 | 0.276819 |
| Ego52 | Tgu11 | 0.325607 |
| Ase24 | Tgu11 | 0.204872 |
| BF18 | Tgu11 | 0.426137 |
| Ind37 | Tgu11 | 0.340764 |
| Ego15 | Cuu4 | 0.108394 |
| Ego26 | Cuu4 | 0.175472 |
| Ego31 | Cuu4 | 0.349392 |
| Ego34 | Cuu4 | **0.041076** |
| Ego49 | Cuu4 | 0.730567 |
| Ego52 | Cuu4 | **0.035861** |
| Ase24 | Cuu4 | 0.559942 |
| BF18 | Cuu4 | 0.423064 |
| Ind37 | Cuu4 | 0.519046 |
| Ego15 | Cuu4 | 0.516717 |
| Ego15 | Ind28 | 0.620335 |
| Ego26 | Ind28 | 0.932433 |
| Ego31 | Ind28 | 0.727195 |
| Ego34 | Ind28 | 0.896817 |
| Ego49 | Ind28 | 0.083154 |
| Ego52 | Ind28 | 0.357335 |
| Ase24 | Ind28 | 0.064868 |
| BF18 | Ind28 | 0.120058 |
| Ind37 | Ind28 | 0.548723 |
| Ego15 | Ind28 | 0.669828 |
| Ego15 | Ind28 | 0.430285 |
| Ego15 | Ind41 | 0.536852 |
| Ego26 | Ind41 | **0.000541** |
| Ego31 | Ind41 | 0.61758 |
| Ego34 | Ind41 | 0.261592 |
| Ego49 | Ind41 | 0.796281 |
| Ego52 | Ind41 | 0.236599 |
| Ase24 | Ind41 | 0.617847 |
| BF18 | Ind41 | 0.674755 |
| Ind37 | Ind41 | 0.095534 |
| Ego15 | Ind41 | 0.769595 |
| Ego15 | Ind41 | 0.901013 |
| Ego15 | Ind41 | 0.330448 |
| Ego15 | Pca7 | 0.948381 |
| Ego26 | Pca7 | 0.519102 |
| Ego31 | Pca7 | 0.215805 |
| Ego34 | Pca7 | 0.465952 |
| Ego49 | Pca7 | 0.374468 |
| Ego52 | Pca7 | 0.098997 |
| Ase24 | Pca7 | 0.553526 |
| BF18 | Pca7 | 0.434907 |
| Ind37 | Pca7 | **0.029283** |
| Tgu11 | Pca7 | 0.330017 |
| Cuu4 | Pca7 | **0.041938** |
| Ind28 | Pca7 | 0.746738 |
| Ind41 | Pca7 | 0.361844 |
| Ego15 | Pco2 | 0.2242 |
| Ego26 | Pco2 | 0.662943 |
| Ego31 | Pco2 | 0.516495 |
| Ego34 | Pco2 | 0.524357 |
| Ego49 | Pco2 | 0.095903 |
| Ego52 | Pco2 | 0.258396 |
| Ase24 | Pco2 | 0.41678 |
| BF18 | Pco2 | 0.972699 |
| Ind37 | Pco2 | 0.678384 |
| Tgu11 | Pco2 | 0.490333 |
| Cuu4 | Pco2 | **0.023427** |
| Ind28 | Pco2 | 0.668428 |
| Ind41 | Pco2 | **0.047054** |
| Pca7 | Pco2 | 0.130533 |
| Ego15 | Titgata02 | 0.583406 |
| Ego26 | Titgata02 | 0.74044 |
| Ego31 | Titgata02 | **0.008375** |
| Ego34 | Titgata02 | 0.764913 |
| Ego49 | Titgata02 | 0.154271 |
| Ego52 | Titgata02 | 0.265703 |
| Ase24 | Titgata02 | 0.520606 |
| BF18 | Titgata02 | 0.280592 |
| Ind37 | Titgata02 | 0.678128 |
| Tgu11 | Titgata02 | 0.945373 |
| Cuu4 | Titgata02 | 0.458788 |
| Ind28 | Titgata02 | 0.29573 |
| Ind41 | Titgata02 | 0.104625 |
| Pca7 | Titgata02 | 0.300377 |
| Pco2 | Titgata02 | 0.096827 |

**References:**

Dawson DA, Hanotte O, Greig C, Stewart IRK, Burke T (2000) Polymorphic microsatellites in the blue tit Parus caeruleus and their cross-species utility in 20 songbird families. *Molecular Ecology*, **9**, 1941–1944.

Excoffier LG, Lischer HEL (2010) Arlequin suite ver 3.5: a new series of programs to perform population genetic analyses under Linux and Windows. *Molecular Ecology Resources*, **10**, 564–567.

Forstmeier W, Holger S, Schneider M, Kempenaers B (2007) Development of polymorphic microsatellite markers for the zebra finch (Taeniopygia guttata). *Molecular Ecology Notes*, **7**, 1026–1028.

Gibbs HL, De Sousa L, Marchetti K, Nakamura H (1998) Isolation and characterization of microsatellite DNA loci for an obligate brood parasitic bird, the common cuckoo (Cuculus canorus). *Molecular ecology*, **7**, 1437–1439.

Kalinowski ST, Taper ML, Marshall TC (2007) Revising how the computer program CERVUS accommodates genotyping error increases success in paternity assignment. *Molecular ecology*, **16**, 1099–106.

Kim K, Zhang H, Horsburgh GJ *et al.* (2015) Four-way development of microsatellite markers for the Gouldian finch (Erythrura gouldiae). *Conservation Genetics Resources*, **7**, 899–907.

Richardson DS, Jury FL, Dawson DA *et al.* (2000) Fifty Seychelles warbler (Acrocephalus sechellensis) microsatellite loci polymorphic in Sylviidae species and their cross-species amplification in other passerine birds. *Molecular Ecology*, **9**, 2225–2230.

Saito D, Nishiumi I, Nakamura M (2001) Characterization of nine polymorphic microsatellite loci from the alpine accentor Prunella collaris. *Molecular Ecology Notes*, **1**, 258–259.

Sefc KM, Payne RB, Sorenson MD (2001) Characterization of microsatellite loci in village indigobirds Vidua chalybeata and cross-species amplification in estrildid and ploceid finches. *Molecular Ecology Notes*, **1**, 252–254.

Wang M, Hsu Y, Yao C, Li S-H (2005) Isolation and characterization of 12 tetranucleotide repeat microsatellite loci from the green-backed tit (Parus monticolus). *Molecular Ecology Notes*, **5**, 439–442.

Yodogawa Y, Nishiumi I, Saito D, Okanoya K (2003) Characterization of eight polymorphic microsatellite loci from the Bengalese finch (Lonchura striata var. domestica). *Molecular Ecology Notes*, **3**, 183–185.
