# Supplementary material for: Three Molecular Markers Show No Evidence of Population Genetic Structure in the Gouldian Finch (Erythrura gouldiae)
Source: PLoS One. 2016 Dec 9;11(12):e0167723. doi: 10.1371/journal.pone.0167723 (PMC5147959; doi:10.1371/journal.pone.0167723)
Supplement: S3 Appendix — Figures and tables include uncorrected p-values for differences between each population comparison, pairwise estimates of genetic differentiation and spatial autocorrelation. (DOCX) [file pone.0167723.s003.docx]

**S3 Appendix: Pairwise Diversity Differentiation & Spatial Autocorrelation Results**

|  | **Mornington** | **Wyndham** | **Bradshaw** | **Delamere** | **Yinberrie Hills** |
| --- | --- | --- | --- | --- | --- |
| **Mornington** |  | 0.574 | 0.360 | 0.578 | **0.036** |
| **Wyndham** | 0.53 |  | 0.680 | 0.583 | 0.345 |
| **Bradshaw** | 0.63 | 1 |  | 0.765 | 0.502 |
| **Delamere** | 0.71 | 0.82 | 0.86 |  | 0.579 |
| **Yinberrie Hills** | 0.32 | 0.63 | 0.82 | 0.82 |  |
| **Chidna** | 0.32 | 0.53 | 0.53 | 0.40 | 0.40 |

**Table A**: P-values for pairwise Wilcox sign-rank tests on microsatellite allelic richness between each of the sampling localities. Below the diagonal reflects the difference in richness on the data rarefied to match the sample size of Chidna. Above the diagonal, is the pairwise p-values between the five major sampling localities of the same sample size. Bolded P-values are significant before Bonferroni correction, but none were significant after Bonferroni correction.

**Table B:** P-values for pairwise Wilcox sign-rank tests on microsatellite heterozygosities between each of the sampling localities. Below the diagonal is the difference in observed heterozygosity. Above the diagonal is for expected heterozygosity. All P-values presented are the raw (not Bonferroni corrected) values, but none were significant before or after correction.

|  | **Mornington** | **Wyndham** | **Bradshaw** | **Delamere** | **Yinberrie Hills** | **Chidna** |
| --- | --- | --- | --- | --- | --- | --- |
| **Mornington** |  | 0.90 | 1.00 | 0.94 | 0.94 | 0.46 |
| **Wyndham** | 0.83 |  | 0.78 | 0.86 | 0.80 | 0.78 |
| **Bradshaw** | 0.74 | 0.84 |  | 0.63 | 0.94 | 0.82 |
| **Delamere** | 0.59 | 0.53 | 0.38 |  | 0.98 | 0.60 |
| **Yinberrie Hills** | 0.86 | 0.93 | 0.75 | 0.31 |  | 0.94 |
| **Chidna** | 0.59 | 0.78 | 0.60 | 0.86 | 0.52 |  |

|  | **Mornington** | **Wyndham** | **Bradshaw** | **Delamere** | **Yinberrie Hills** | **Chidna** |
| --- | --- | --- | --- | --- | --- | --- |
| **Mornington** |  | 0.0078 | **0.0286** | 0.0158 | 0.0215 | 0.0268 |
| **Wyndham** | 0.0006 |  | **0.0198** | 0.0112 | 0.0097 | 0.0392 |
| **Bradshaw** | **0.0042** | 0.0018 |  | **0.0287** | 0.0000 | 0.0000 |
| **Delamere** | 0.0020 | 0.0012 | 0.0029 |  | 0.0003 | 0.0000 |
| **Yinberrie Hills** | 0.0024 | 0.0003 | 0.0000 | 0.0000 |  | 0.0247 |
| **Chidna** | 0.0139 | 0.0116 | 0.0040 | 0.0009 | 0.0074 |  |

**Table C:** Estimates of differentiation (F_ST_ and Jost’s D) from sixteen microsatellite markers from the six sampling localities. Below the diagonal is pairwise F_ST_, where p-values are bolded at p<0.05, but no comparison was significant after Bonferroni correction (p<0.0051). Above the diagonal are Jost’s D, emboldened are p-values calculated by 1000 bootstrap resamples in DEMEtics, but none were significant after Bonferroni correction. Negative F_ST_ and D estimates are corrected to zero.

**Table D:** Pairwise F-ST based on mitochondrial control region haplotype frequencies (below diagonal); Pairwise Φst based on nucleotide diversity in each population using the Kimura 2 Parameter model above the diagonal. Values in bold are significant at p=0.05, but no comparison was significant after Bonferroni correction (p<0.0033). All negative F_ST_ results were corrected zero.

|  | **Mornington** | **Wyndham** | **Bradshaw** | **Delamere** | **Yinberrie Hills** | **Chidna** |
| --- | --- | --- | --- | --- | --- | --- |
| **Mornington** |  | 0.00000 | 0.00000 | 0.00000 | 0.00924 | **0.19371** |
| **Wyndham** | 0.01233 |  | 0.00000 | 0.00000 | 0.02324 | **0.33265** |
| **Bradshaw** | 0.00570 | 0.0000 |  | 0.00000 | 0.00000 | **0.15414** |
| **Delamere** | 0.00127 | 0.0000 | 0.00000 |  | 0.01939 | **0.30028** |
| **Yinberrie Hills** | 0.03224 | 0.0000 | 0.00000 | 0.00000 |  | 0.09665 |
| **Chidna** | **0.15141** | **0.26941** | **0.16275** | **0.20683** | **0.19471** |  |

**Table E:** Below diagonal: Pairwise F_ST_ from Arlequin resulting from 3839 SNP dataset, negative values are corrected to zero. Significant differences (p<0.05) are emboldened, but after Bonferonni correction (p<0.0033), no result was statistically significant against 10100 permutations.

|  | **Mornington** | **Wyndham** | **Bradshaw** | **Delamere** | **Yinberrie Hills** |
| --- | --- | --- | --- | --- | --- |
| **Mornington** |  |  |  |  |  |
| **Wyndham** | 0.000 |  |  |  |  |
| **Bradshaw** | 0.000 | 0.000 |  |  |  |
| **Delamere** | 0.000 | 0.000 | 0.000 |  |  |
| **Yinberrie Hills** | 0.000 | 0.000 | 0.000 | 0.000 |  |
| **Chidna** | 0.007 | **0.011** | 0.005 | 0.009 | 0.005 |

**
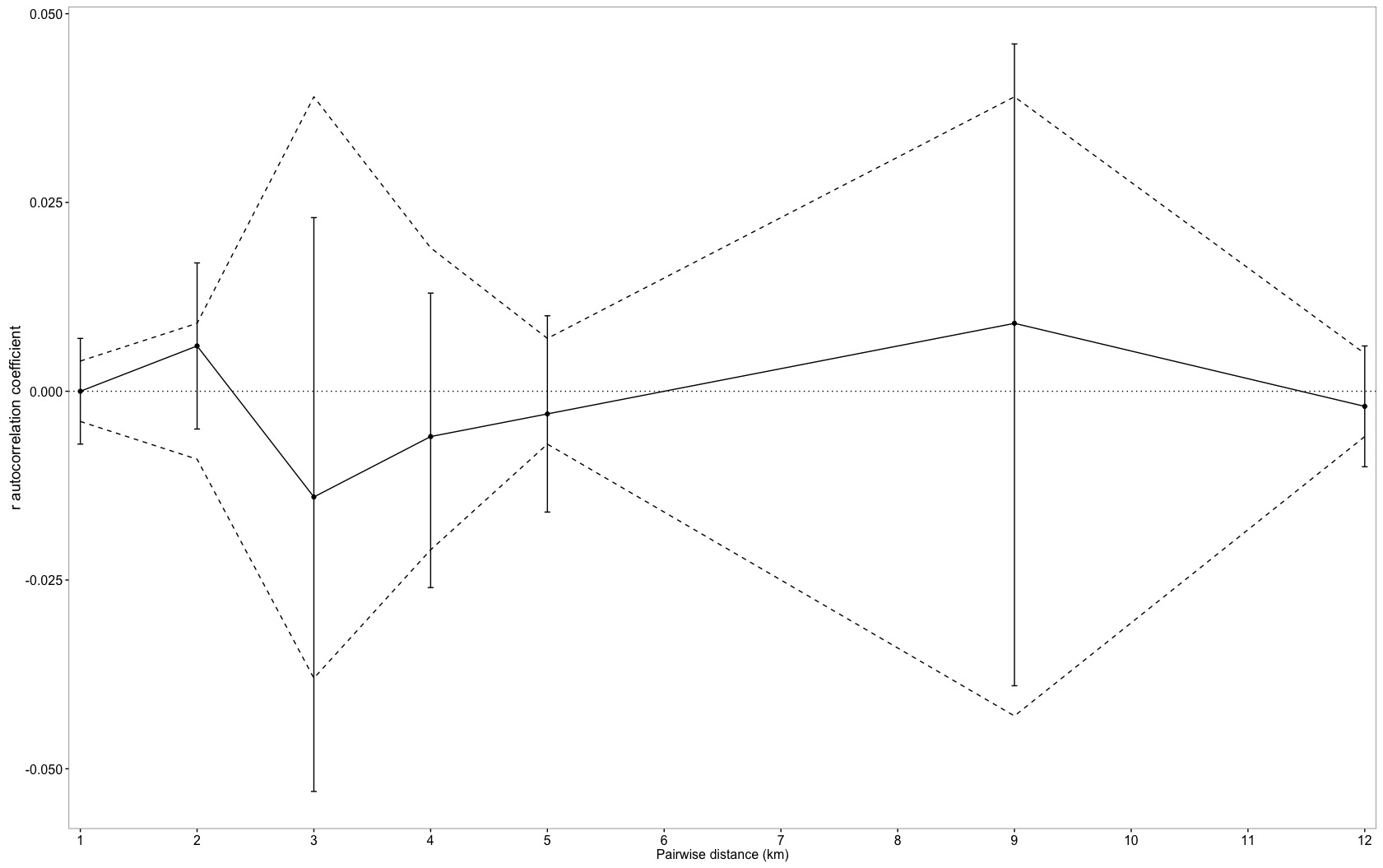
**

**Figure A:** Correlogram from spatial autocorrelation analysis, showing no spatial structure at the local scale within the Wyndham population. As there were no sex differences only the pooled results are presented. The unbroken line (r) represents the autocorrelation coefficient from the comparison between genetic and geographic distance matrices, and error bars are the bootstrap 95% confidence intervals about the *r* estimate for that distance class. The dashed lines represent the upper and lower 95% confidence intervals for the null hypothesis of no spatial structure (around r=0, dotted line) generated by permutation of samples across the distance classes
