# Supplementary material for: Three Molecular Markers Show No Evidence of Population Genetic Structure in the Gouldian Finch (Erythrura gouldiae)
Source: PLoS One. 2016 Dec 9;11(12):e0167723. doi: 10.1371/journal.pone.0167723 (PMC5147959; doi:10.1371/journal.pone.0167723)
Supplement: S4 Appendix — Text and figures describe the detailed methods and model checks associated with the analysis of the microsatellite and SNP data in adegenet. (DOCX) [file pone.0167723.s004.docx]

**S4 Appendix: K-means clustering and Discriminant Analysis of Principal Components in ‘*adegenet*’**

We followed the recommendations of the developers using the the dapc tutorial online (Jombart & Collins 2015)

*Discriminant Analysis on microsatellite dataset:*

In order to infer the number of populations without *a priori* sampling information, we ran the function *find.clusters* in R-package adegenet (Jombart 2008) on the dataset with 49 randomly subsampled individuals per population plus six from Chidna. This function turns the original genotypic data into uncorrelated principal components, then scores the clustering solutions for different numbers of clusters using the Bayesian Information Criterion. Because we found the optimum number of clusters to be one (K=1) (Figure A), we only proceeded with Discriminant Analysis of Principal Components (DAPC, function *dapc*) using the sampling locality as a prior (Jombart *et al.* 2010).

To ensure the discriminant analysis with population priors was not overfit by retaining too many principal components we ran two model checks using a “full” model including all principal components, linear discriminants among the optimal four clusters identified previously (Jombart *et al.* 2010).

On this full model we ran two procedures that maximises the ability to assign individuals to clusters reliably. Function *optim.a.score* is essentially a permutation test, which maximises the “a-score”, by comparing the number of assignments to “real” number of clusters to randomised clusters. The a-score is a measure the proportion of assignments back to the prior clusters or to random clusters, and is essentially a measure of ‘over-fitting’ of the model. We ran 100 simulations per increase of 16 principal components.

Function *xvalDapc* also attempts to optimise genuine assignment power over random assignments, and does so by cross-validating against training and test subsets of data. We ran this using the default settings which uses 90% of the data as a training dataset, and tested on the remaining 10%. The a-score optimisation showed that no number of retained principal components gives a high assignment success, but there is an elevated success rate between when retaining 80 to 160 principal components (maximum=127) (Figure Bii). The cross-validation procedure showed equal assignment success across all principal components. For the final discriminant analysis we selected 80 principal components, because adegenet also recommends retaining principal components that are less than the number of individuals divided by three (Jombart 2008; Jombart and Collins, 2015).

The final model using 80 retained principal components revealed considerable overlap between individuals from different sampling localities (Figure Ai), and the final model was able to reassign 59% of individuals back to the original sampling population, but the a-score for this configuration was very low (<5%). The poor a-score results and the optimisation procedures carried out suggest it is virtually impossible to find a model that does not over-fit the data. Therefore, sampling locality does not have an important effect on the clustering of this data.

We then proceeded with the *dapc* analysis using sampling locality as a prior, retaining 80 principal components and all linear discriminant functions, as in the microsatellite analysis. These results show that all western populations are clustered together, with some separation evident in the Eastern population at Chidna (Figure S4c).


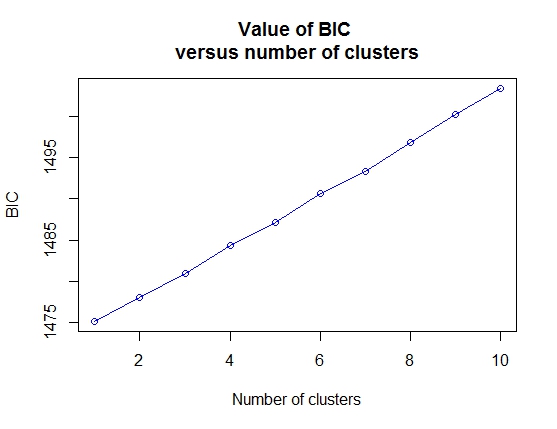


**Figure A:** Results of K-means clustering method *find.clusters* for microsatellite data, with each clustering solution and its corresponding BIC score.


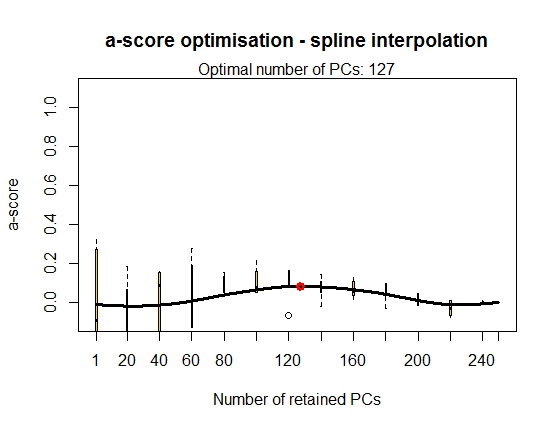

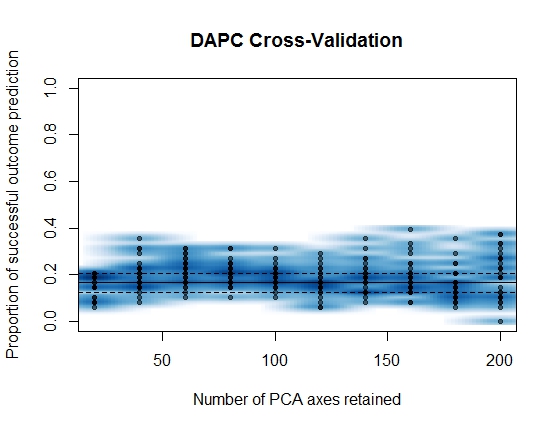

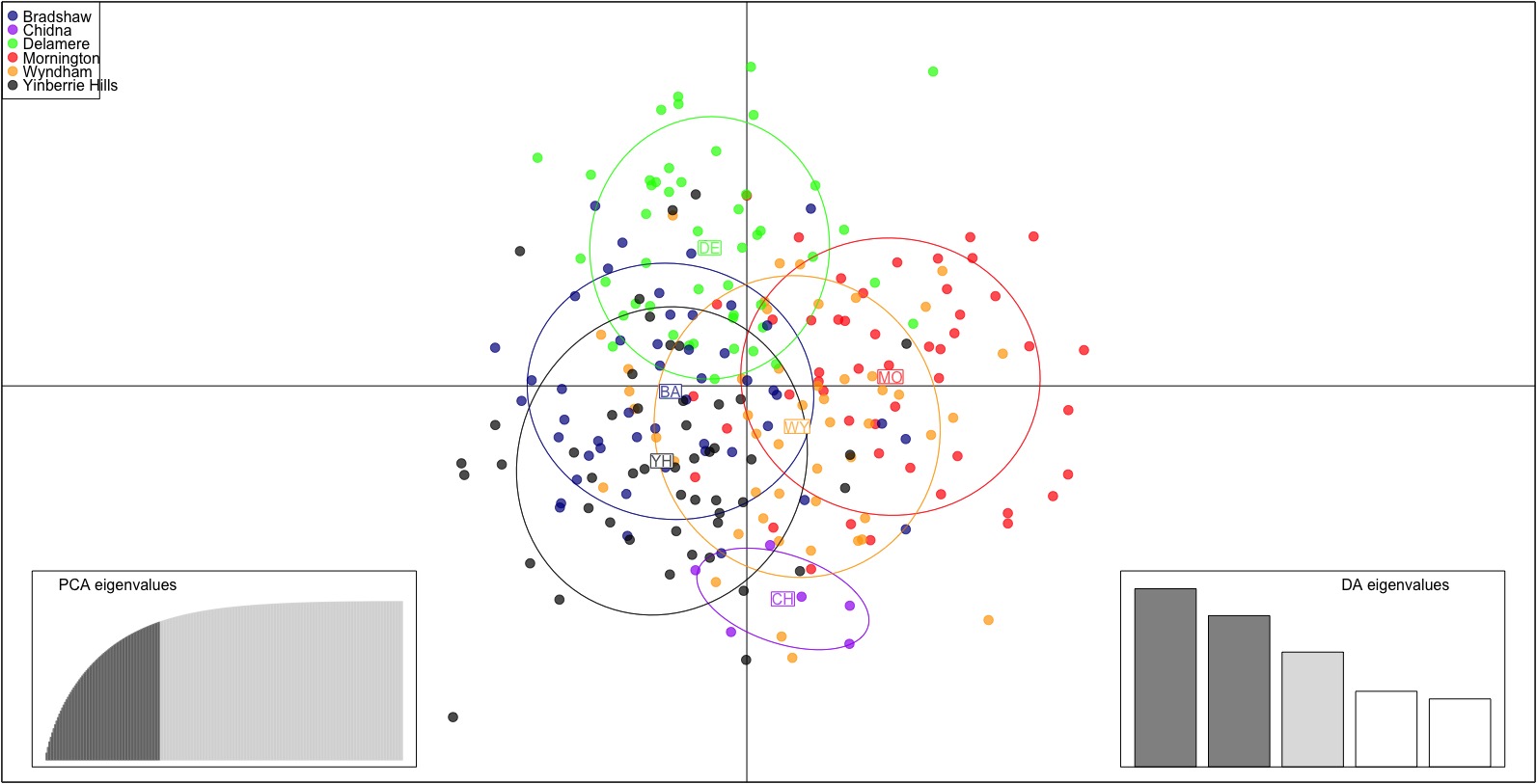
**Figure B**: Discriminant Analysis of Principal Components (DAPC) model optimisation for the best number of principal components of microsatellite data given the sampling localities. Part i) shows the results from the a-score optimisation procedure and ii) shows the results from the cross-validation analysis. Part iii) is the scatterplot of the final DAPC model with sampling locality as a prior, where points are individual genotypes, colour-coded by their original sampling locality and surrounded by a 95% confidence ellipse. DA and PCA Eigenvalues represent the amount of genetic variation captured by the analysis, and the first two discriminant factors are plotted as the x- and y- axis.

iii)

i)

ii)

*Discriminant analysis on SNP dataset:*

The *adegenet* package is also able to run the same analyses on SNP datasets, and essentially ran the same procedure as above. Unlike the microsatellite dataset, the contribution to the total variance per additional principal component did not reach an asymptote, and remained roughly linear (Figure C). Further, in finding the best clustering solution, here was a roughly linear increase in BIC with increase in number of clusters (Figure D), with the lowest BIC at a single cluster. Given *find.clusters* suggested that one cluster was the optimal configuration we did not proceed with DAPC from the *find.clusters* result.

Like the microsatellite data, we explored how the genetic variation was partitioned among our sampling localities using it as a grouping prior in dapc. First we validated the best number of principal components to use using *optim.a.score* function, which was unable to identify an optimal number of PCs to retain (Figure Ei) We then ran a cross-validation analysis as in the microsatellite dataset, but was again unable to find a number of principal components that correctly predict the group membership of clusters (Figure Eii). We then proceeded with the *dapc* analysis using sampling locality as a prior, retaining 80 principal components and all linear discriminant functions, as in the microsatellite analysis. These results show that all western populations are clustered together, with some separation evident in the Eastern population at Chidna (Main text Figure 4).


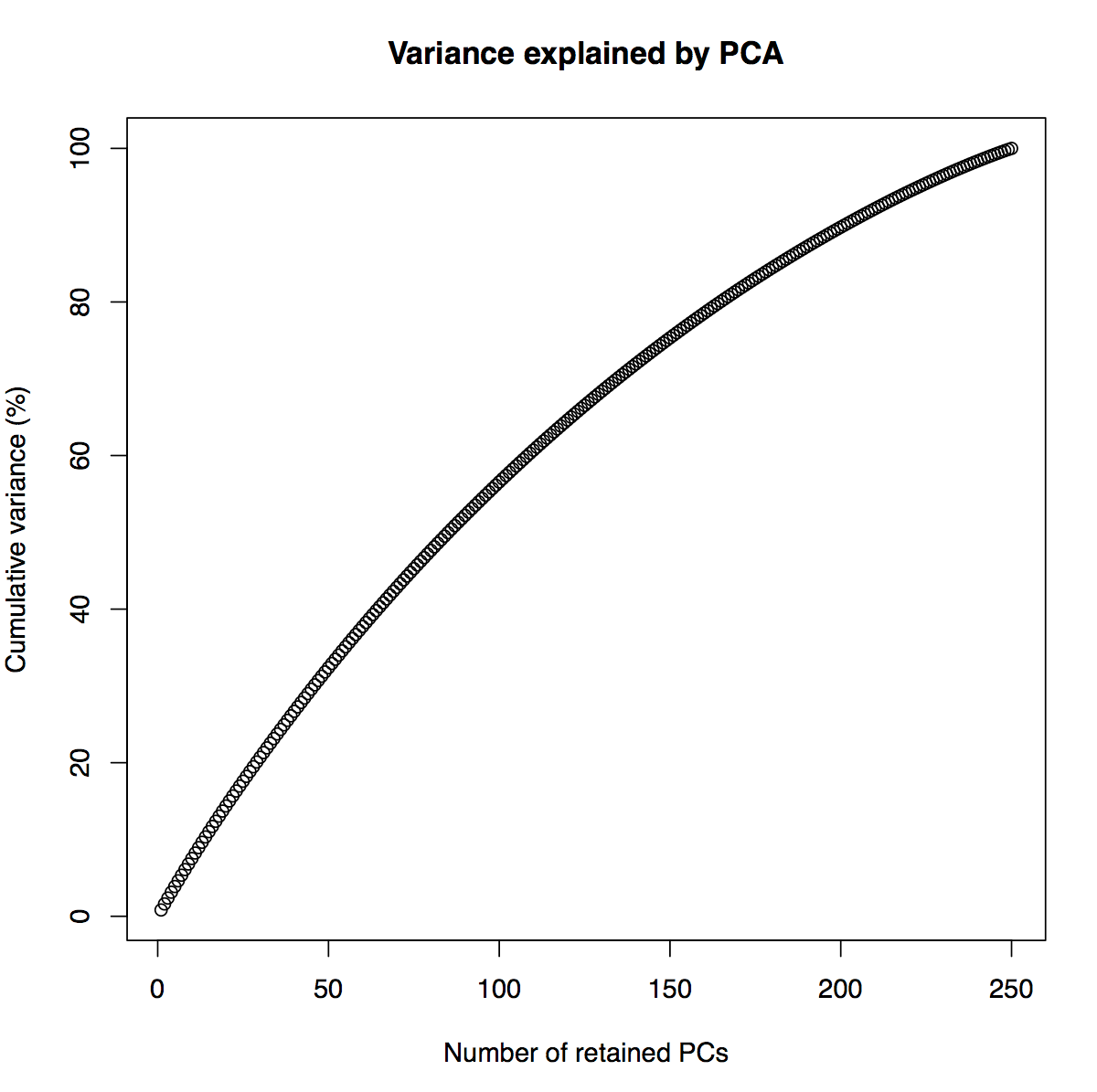


**Figure C:** How the principal components of the SNP dataset represent the variance within the dataset.

**
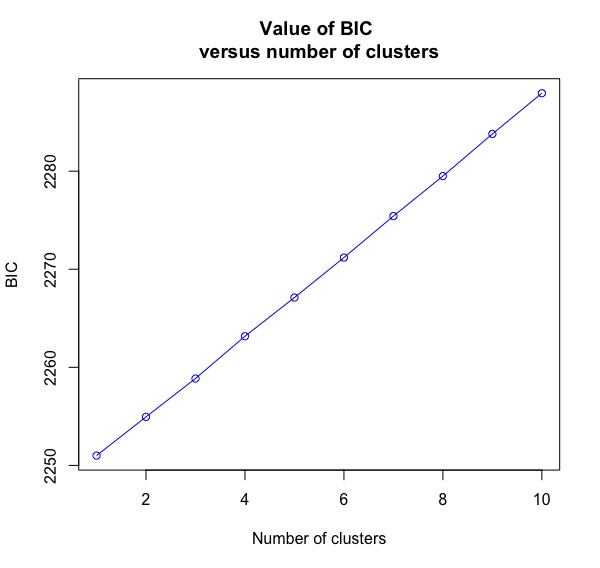
**

**Figure D:** results of *find.clusters* analysis on SNP data, with each clustering solution given a BIC score.


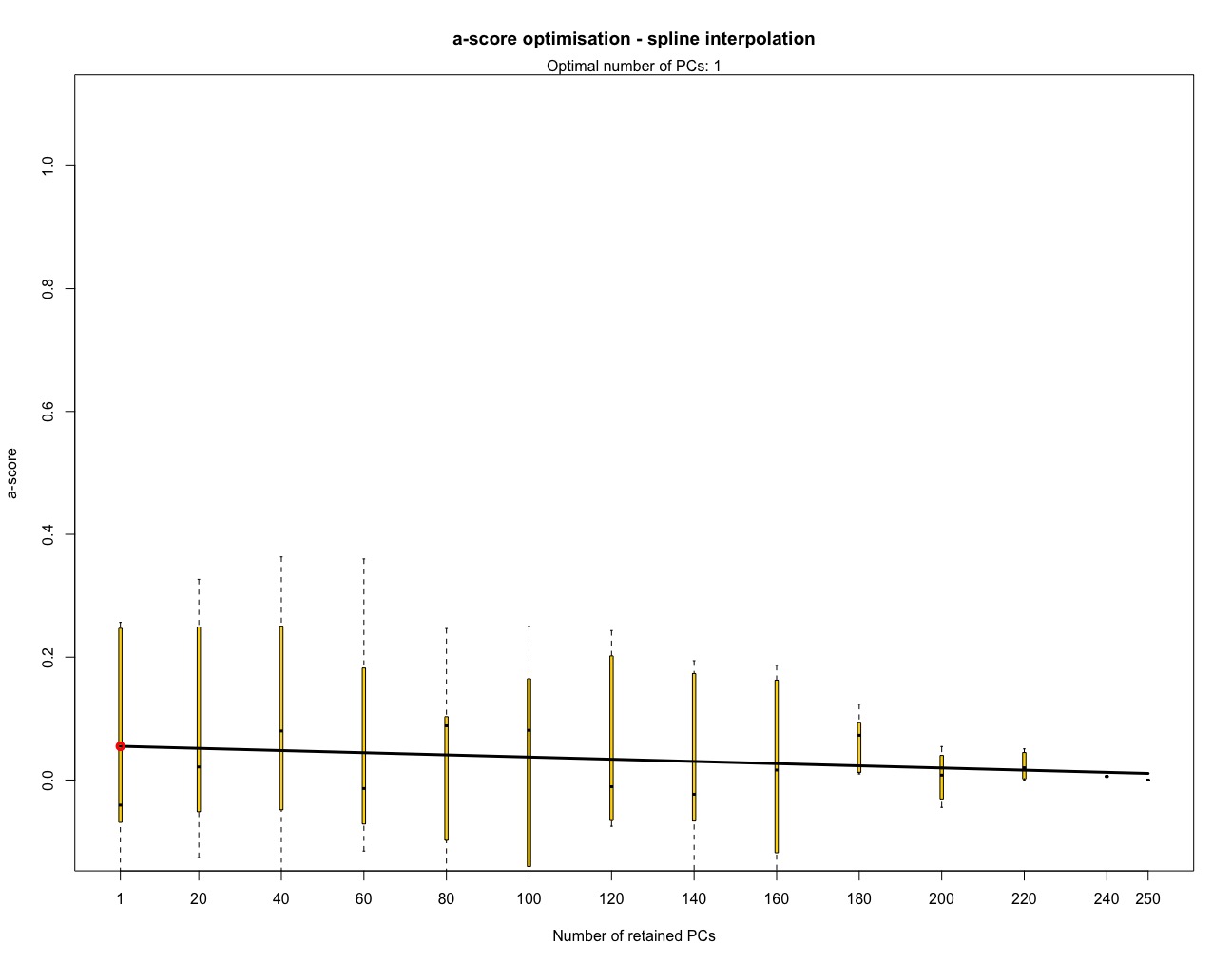

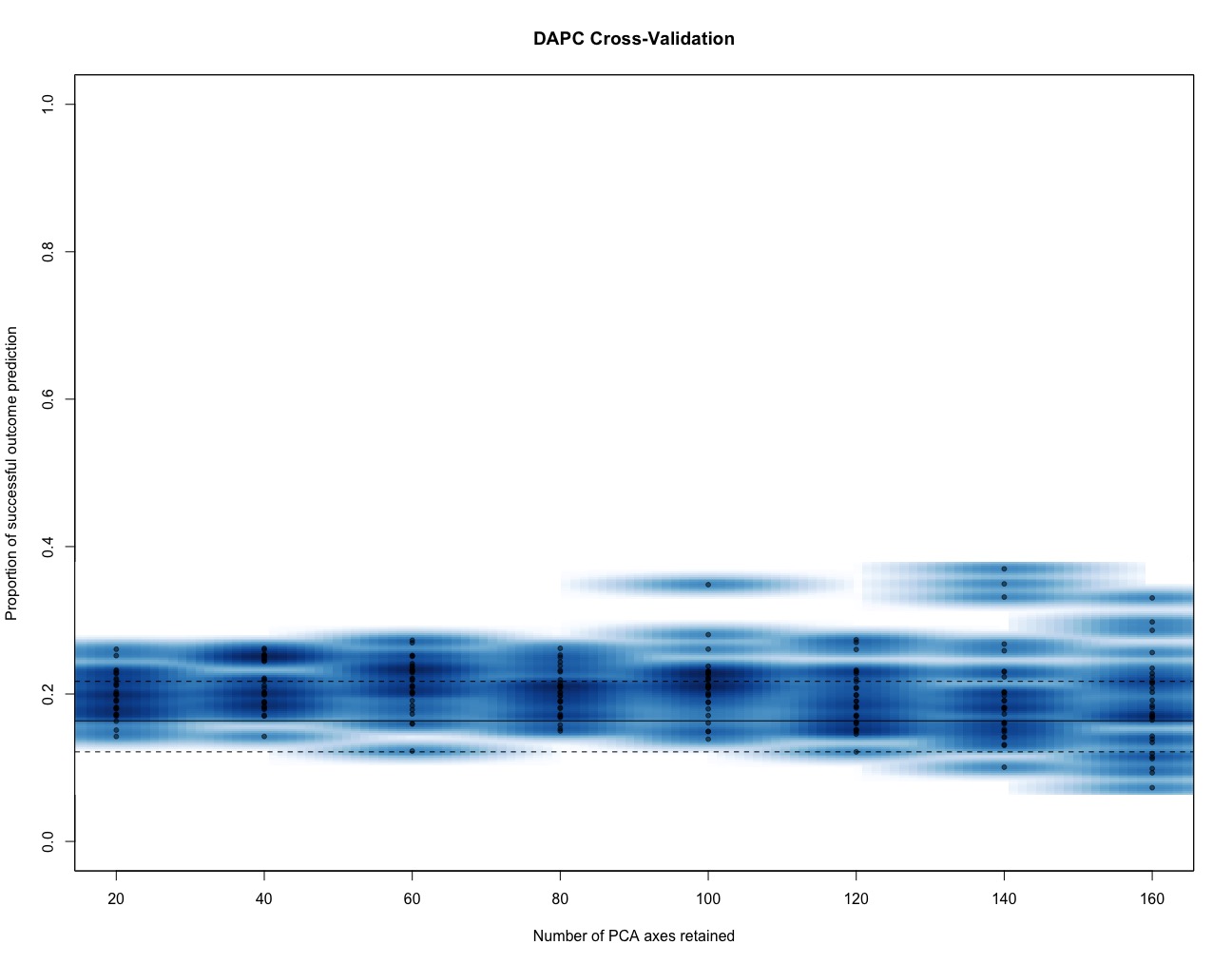


i)

ii)

**Figure E:** Results of i) a-score optimisation on the SNP dataset, and ii) the cross-validation analysis on SNP data

**References:**

Jombart T (2008) adegenet: a R package for the multivariate analysis of genetic markers. *Bioinformatics*, **24**, 1403–1405.

Jombart T, Collins C (2015) A tutorial for Discriminant Analysis of Principal Components (DAPC) using adegenet 2.0.0.
http://adegenet.r-forge.r-project.org/files/tutorial-dapc.pdf

Jombart T, Devillard S, Balloux F (2010) Discriminant analysis of principal components: a new method for the analysis of genetically structured populations. *BMC genetics*, **11**, 94.
